# Diffusive Dynamics of Bacterial Proteome as a Proxy of Cell Death

**DOI:** 10.1021/acscentsci.2c01078

**Published:** 2023-01-09

**Authors:** Daniele Di Bari, Stepan Timr, Marianne Guiral, Marie-Thérèse Giudici-Orticoni, Tilo Seydel, Christian Beck, Caterina Petrillo, Philippe Derreumaux, Simone Melchionna, Fabio Sterpone, Judith Peters, Alessandro Paciaroni

**Affiliations:** † Università degli Studi di Perugia, Dipartimento di Fisica e Geologia, Via A. Pascoli, 06123Perugia PG, Italy; ‡ Université Grenoble Alpes, CNRS, Laboratoire Interdisciplinaire de Physique, 38400Saint-Martin-d’Héres, France; § Institut Laue-Langevin, 38000Grenoble, France; ∥ Laboratoire de Biochimie Théorique (UPR9080), CNRS, Université de Paris Cité, 13 Rue Pierre et Marie Curie, 75005Paris, France; ⊥ Institut de Biologie Physico-Chimique, Fondation Edmond de Rothschild, 13 Rue Pierre et Marie Curie, 75005Paris, France; # J. Heyrovský Institute of Physical Chemistry, Czech Academy of Sciences, 182 23Prague 8, Czechia; ○ Laboratoire de Bioénergétique et Ingénierie des Protéines, BIP, CNRS, Aix-Marseille Université, 13400Marseille, France; □ ISC-CNR, Dipartimento di Fisica, Università Sapienza, 00185Rome, Italy; △ Lexma Technology1337 Massachusetts Avenue, Arlington, Massachusetts02476, United States; ▽ Institut Universitaire de France, 75005Paris, France

## Abstract

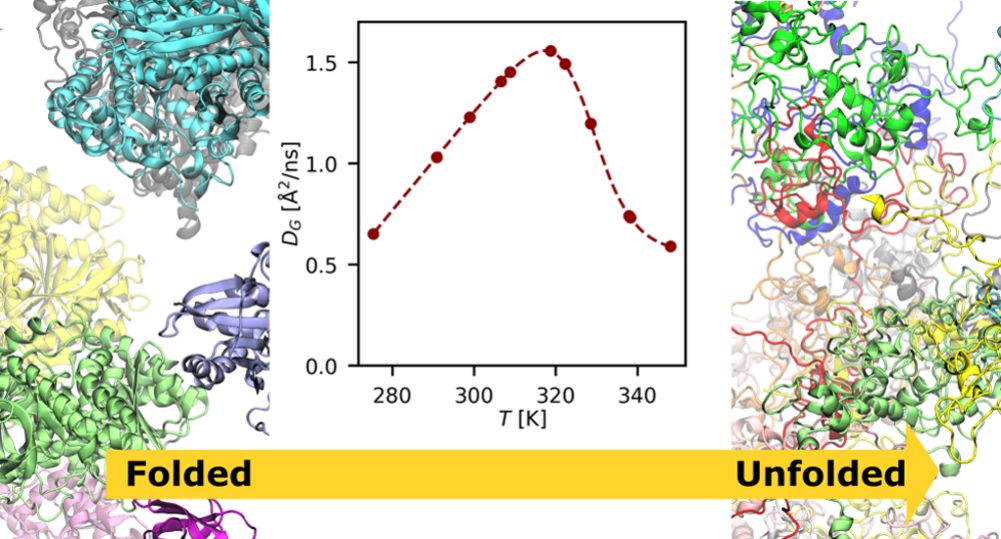

Temperature variations have a big impact on bacterial metabolism and death, yet an exhaustive molecular picture of these processes is still missing. For instance, whether thermal death is determined by the deterioration of the whole or a specific part of the proteome is hotly debated. Here, by monitoring the proteome dynamics of *E. coli*, we clearly show that only a minor fraction of the proteome unfolds at the cell death. First, we prove that the dynamical state of the *E. coli* proteome is an excellent proxy for temperature-dependent bacterial metabolism and death. The proteome diffusive dynamics peaks at about the bacterial optimal growth temperature, then a dramatic dynamical slowdown is observed that starts just below the cell’s death temperature. Next, we show that this slowdown is caused by the unfolding of just a small fraction of proteins that establish an entangling interprotein network, dominated by hydrophobic interactions, across the cytoplasm. Finally, the deduced progress of the proteome unfolding and its diffusive dynamics are both key to correctly reproduce the *E. coli* growth rate.

## Abstract 2

The trend of short-time diffusion of the proteome in *E. coli* mirrors that of the bacterial metabolism. At the cell’s death, only a small fraction of the proteome is unfolded.

## Introduction

A deep understanding of the cell’s thermal stability is key to modeling the impact of climate change on microbial organism growth,^1^ establishing theoretical boundaries for life in extreme environments,^2^ and optimizing thermal-based treatments for cancer.^3^ Moreover, biological migrations, extinctions, genetic divergence, and speciation can all be triggered by small changes in environmental temperature.^4−6^

All the single cellular components suffer in various ways from heat. The membrane integrity can be challenged, resulting in the evasion of periplasmic proteins or the entrance of harmful compounds.^7^ Proteins are mandatory for good cellular functioning, but high temperature provokes the loss of structural stability and unfolding. On the other hand, nucleic acids are more stable against thermal stress,^8^ so that their denaturation can be considered to be a minor cause of cell death. As proteins are the most abundant and less stable biomolecules in the cell, their thermal sensitivity has to play a key role in determining the temperature-dependent cellular activities.

Different hypotheses have been proposed to link the degradation of the proteome to the upper limit of the cellular thermal niche, i.e., the cell’s death temperature *T*_CD_, and to quantify the proteome thermal stability.^9−11^ The cell death has been theoretically explained in terms of a proteome catastrophe, where most of the proteins unfold in a narrow range of temperatures near *T*_CD_.^10,12^ This picture has recently been challenged by experimental investigations of *Escherichia coli* lysates and cells, based on different techniques such as limited proteolysis^11^ or thermal proteome profiling,^13^ combined with mass spectrometry. According to these studies, thermal adaptation would result from the preferential stabilization of a subset of proteins, indicating that the heat sensitivity of cells can be explained by a small number of proteins that serve critical physiological roles.

The proteome’s thermal stability is not the only physical determinant of the cell’s growth rate. Indeed, molecular transport and cytoplasmic mixing in bacteria rely largely on diffusive motions, which are therefore considered an integral part of the cell life.^14^ Diffusion is crucial for cell growth by promoting correct spatiotemporal localization of cytoplasmic components and regulating the partition of solutes between daughter cells. In addition, diffusion determines the mobility of cytoplasmic constituents and hence sets the boundaries at which some key molecular interactions (and the relevant biological reactions) can occur. For instance, prototypical cases of diffusion-limited biological reactions, i.e., reactions where the slowest step is the coming together of proteins, are the ones that involve protein pairs such as Barnase–Barstar from *Bacillus amyloliquefaciens*.^15^ The efficient binding of Barnase to Barstar in the cytoplasm, which is key to prevent damage of endogenous RNA, is likely due to a large diffusion-limited on-rate constant, *k*_on_.^16^ In addition, critical cellular processes would not occur at all in the absence of specific protein diffusion coefficients, as in the case of the oscillation of the Min system, which ensures proper spatial and temporal regulation of chromosomal segregation during the *E. coli* cellular division.^14^ Diffusion of proteins in turn depends on temperature, and beyond a critical value, their unfolding might lead to a drastic change of the environmental viscosity and, as already observed in crowded solutions,^17−19^ to the slowdown of their mobility.

To date, the relationship between the diffusive dynamics of the proteome and the thermal sensitivity of a cell has not yet been investigated in the temperature range including the cell death. It is an extremely difficult task to extract the motions of proteins in the crowded milieu of cell’s cytoplasm, where the local protein concentration may vary from 200 up to 400 g/L.^20^ Here, the protein diffusive dynamics is affected by several factors, such as the presence of steric barriers given by other macromolecules, hydrodynamic and attractive interactions, and spatial heterogeneity, all of which can vary as a function of the cell’s physiological state and metabolic activity.^21−24^

On these grounds, we provide an unprecedented picture of the dynamics of the *E. coli*’s proteome in the nanosecond time-scale, based on state-of-the-art neutron scattering spectroscopy and multiscale molecular dynamics simulations. We show that in *E. coli* the global protein diffusion is a close proxy of the bacterial metabolism, with a linear Stokes–Einstein dependence in the lower temperature range and a striking dynamic slowdown above the thermal death. By connecting the information on the proteome’s dynamics obtained from neutron scattering and simulations, we quantitatively describe the way the unfolded protein fraction progressively increases with temperature, and how this alters the physical–chemical environment of the cytoplasm.

We clearly show that no global proteome unfolding occurs at cell death. Finally, we combine the results from these two approaches and verify that the derived proteome stability curve and temperature-dependent proteome diffusivity together allow to perfectly reproduce the *E. coli* growth rate profile.

## Results

### Neutron Scattering Shows a Dramatic Slowdown of the *E. coli* Proteome Diffusive Dynamics

State-of-the-art Quasi-elastic Neutron Scattering (QENS) experiments were performed on the backscattering spectrometer IN16b^25^ at the Institut Laue Langevin (ILL), France, on in vivo *E. coli* samples. QENS allows to directly measure the nanosecond time scale dynamics of the investigated system, through the incoherent dynamic structure factor *S*(*Q*, *E*).^26^ The scattering signal of these samples is mainly due to the large incoherent scattering cross section of the hydrogen atoms, which report mainly on the self-diffusive dynamics of the average protein in the bacterial cytoplasm.^27,28^ The dynamical state of the proteome was sampled at increasing temperatures starting from 276 K, where the bacteria can live and thrive, until 350 K, i.e., well above the temperature of cell-death (*T*_CD_ ≈ 323 K).^10^ In addition, to test the reversibility of dynamical changes, we performed a few measurements while cooling the bacteria after they underwent thermal death.

Protein dynamics at all the temperatures are well described in terms of two distinct diffusive processes arising from the global (G) and local (L) dynamics of the average protein in the *E. coli* cytoplasm (for further details see SI).

**Figure 1:**
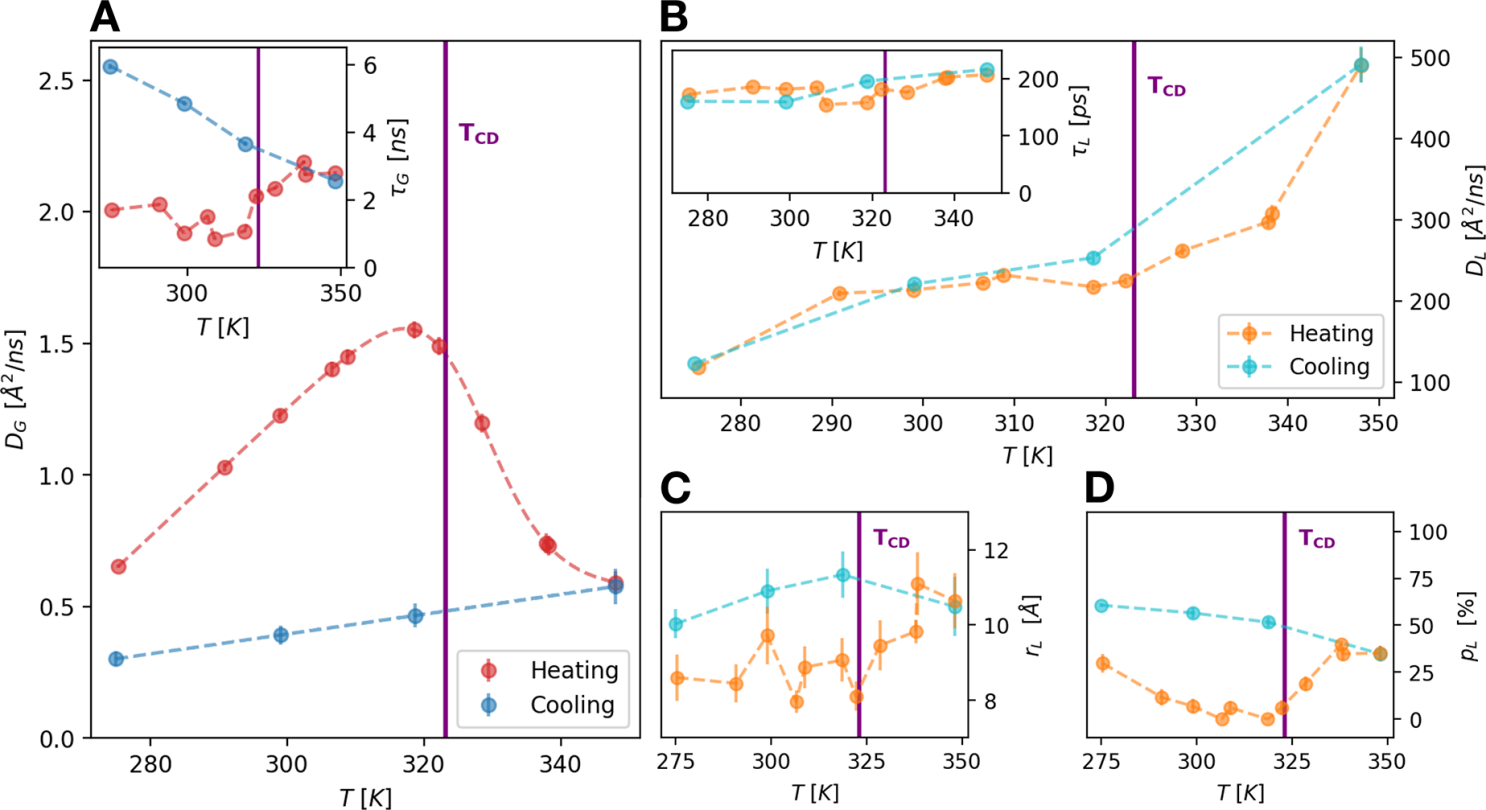
(A) Apparent self-diffusion coefficient, *D*_G_, of an average protein in the *E. coli* cytoplasm as a function of temperature. A clear non reversible slowdown of *D*_G_ is visible after the temperature of cell death (*T*_CD_) due to the gelation of the cytoplasm. The inset shows the residence time τ_G_ for the global motions as a function of temperature that undergoes an important upturn after *T*_CD_ and continues to increase during the cooling. (B) Temperature dependence of the diffusion coefficient *D*_L_ for the local motions of the side-chains. The transition at *T*_CD_, where *D*_L_ starts to increase more steeply with the temperature, is reversible. The inset shows the residence time τ_L_ for the internal dynamics which is nearly constant (≈180 ps). (C, D) Geometries of the local motions as derived by the elastic incoherent structure factor (EISF):^26,30^*r*_*L*_ is the radius of the confinement region for this type of fluctuations (C), and *p*_L_ is the fraction of atoms appearing fixed on the accessible time scale (D).

The derived apparent diffusion coefficient *D*_G_, which combines both the translational and rotational motions of proteins^29^ (see SI) exhibits a dramatic nonreversible reduction in proximity of the cell-death temperature (Figure 1A). In protein crowded solutions a similar slowdown of the diffusive dynamics was ascribed to the gelation of the system induced by the protein unfolding.^17−19^ The transition to a gel-like phase is also supported by the slight increase of τ_G_ above *T*_CD_ (inset of Figure 1A), which suggests an increasing localization of proteins in cage-like structures.

Further, the internal local dynamics of the average protein is very sensitive to temperature change, but in this case the diffusion coefficient *D*_L_ shows a significant increase above *T*_CD_, while τ_L_ is nearly constant at ≈180 ps (Figure 1B and its inset). This suggests that the changes in the local dynamics are due to a variation in the extent of the explored spatial region. This is confirmed by the increase of the distance between two jumps *r*_L_ that displays a sudden break toward higher values after *T*_CD_ (Figure 1C). Interestingly, also the number of atoms too slow to be visible by QENS, *p*_L_, shows a similar increasing trend above *T*_CD_, as seen in Figure 1D, when temperature is rising. This behavior is consistent with the progressive unfolding of a part of the *E. coli* proteome. In fact, moving subgroups of denatured proteins can access larger spatial regions, and at the same time their number gradually decreases because of the growing interactions of the gel-like system formed in the cytoplasm.

The rate at which protein groups locally explore their environment seems to be reversible across the thermal death, as we can see from the trend of both *D*_L_ and τ_L_ in Figure 1B, confirming previous results on crowded protein solutions.^18^ On the other hand, both *r*_L_ and *p*_L_ show an irreversible trend.

### Simulations Show How Protein Unfolding Slows down Protein Diffusion

To examine how protein motions are affected by heating and thermal denaturation, we performed multiscale Molecular Dynamics (MD) simulations. Previous works examined protein diffusion in crowded environments using coarse-grained (CG)^31,32^ and all-atom^33,34^ MD simulations. Here, we combined two levels of description: a CG model for protein (OPEP) owning residue-level chemical resolution^35^ to sample the local structure of the crowded solution and an all-atom description to subsequently explore the diffusion of proteins in subvolumes obtained from the coarse-grained simulation. In our approach we used the Lattice Boltzmann Molecular Dynamics (LBMD) technique, which allows including naturally hydrodynamic interactions in the simulations of implicit solvent CG molecular models,^36^ and was already successfully applied to protein crowded solutions.^37^

**Figure 2:**
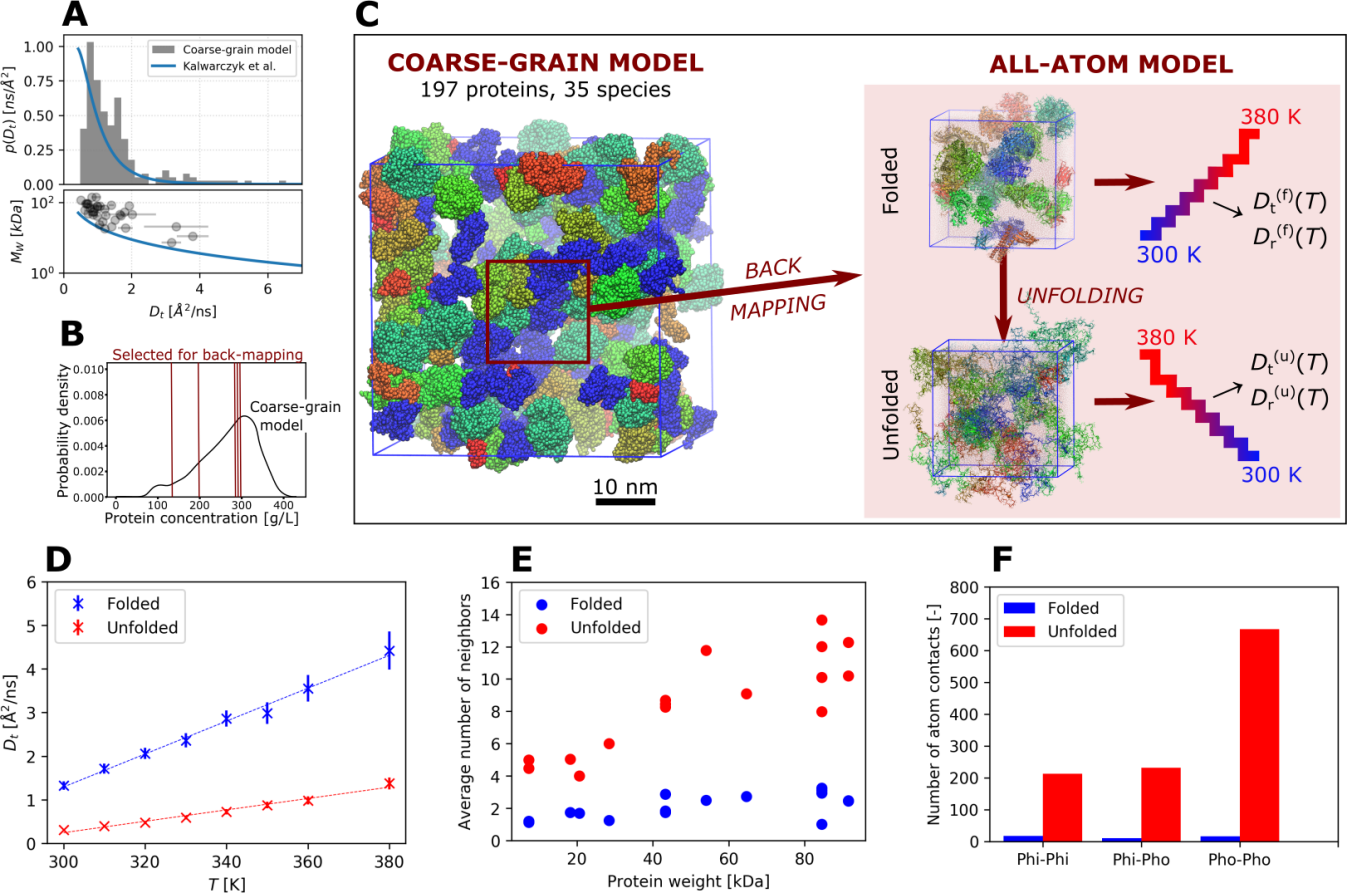
(A) Protein diffusion in the CG model cytoplasm simulated by LBMD. Distribution of the translational diffusion coefficients (top inset), and the dependence of the proteins’ molecular weight on these coefficients (bottom inset). The solid lines represent the fits of the experimental data reported in ref (38). (B) Distribution of local protein concentrations in 17 nm cubic sub-boxes randomly placed in the large simulation system. The vertical lines correspond to the local concentrations of the sub-boxes that were back-mapped at the atomistic resolution. (C) Pictorial representation of the CG cytoplasm system (cubic box of side 40 nm), the schematic strategy of the back-map, and the temperature scans for the folded and unfolded versions of the atomistic systems. (D) Average protein translational diffusion coefficients, computed in the atomistic systems for the 0.3–5 ns regime as a function of temperature. Note that the translational diffusion coefficients were corrected for the effects of periodic boundary conditions, as described in SI. (E) Average number of interaction partners per protein as a function of its molecular weight. (F) Average number of different classes of protein–protein contacts (hydrophilic–hydrophilic (Phi-Phi), hydrophilic–hydrophobic (Phi-Pho), and hydrophobic–hydrophobic (Pho-Pho)) per protein. An atom was considered as “hydrophobic” if its partial charge was less than 0.2 *e* in magnitude. The plots show results obtained with the a99SB-disp force field; the results for CHARMM36m can be found in SI (Figures S11 and S12). The data in panels (E) and (F) were obtained from the production simulations of the folded and unfolded versions of the 288 g/L subvolume at *T* = 330 K.

The 4.3 μs long coarse-grained LBMD simulation contained 197 proteins of 35 different species including chaperon proteins (DnaK) and cold-shock proteins (CspC), mimicking the protein composition of the *E. coli* cytoplasm^32^ (see Figure 2 for a pictorial representation of the system and SI for more details). The length of the trajectory allowed significant reshuffling of the initial positions of the proteins and thus also exploration of different geometries of the crowded system; in fact, only 35% of the initial interaction partners were still in contact at the end of the trajectory (see Figure S9 in SI). The observed reshuffling is associated with a computed diffusivity for each molecular species, very close to its experimental estimate. In Figure 2A we report the distribution of the translational diffusion coefficients computed for each protein. The coefficients vary between 0.5 and 7 Å^2^/ns depending on the molecular weight (see Table S3 in SI), these values being in excellent agreement with experiments^38^ (see blue solid lines in the graphs).

We selected five subvolumes of the whole system containing 11–20 proteins from different frames of this large-scale trajectory to reflect the protein composition and concentration heterogeneity of the cytoplasm (Figure 2B). Each sub-box was converted to the all-atom resolution (Figure 2C) and exposed to a sequence of production simulations (∼100 ns per run) at increasing temperatures, to investigate protein translational diffusion coefficients, probed in the 0.3–5 ns regime. Subsequently, for these atomistic systems we repeated the heating protocol but with the proteins in the unfolded state (see SI).

Our simulations showed a strong decrease of the average translational diffusion coefficient upon unfolding (Figure 2D). The observed decrease quantitatively agreed for the two force fields we used, a99SB-disp^39^ and CHARMM36m^40^ (see Figure S11 in SI for the result obtained with CHARMM36m). In addition, for both folded and unfolded proteins the diffusion coefficient scales linearly with temperature. To be noted that the rotational component gives only a minor contribution to the apparent protein diffusion, with the *D*_t_/*D*_G_ ratio equaling ∼70–90% (see SI).

### Unfolding Changes Protein Interactions

A detailed look at the protein–protein interactions reveals the cause of the diffusion slowdown. We consider as an example one of the simulated atomistic systems of protein concentration 288 g/L. While each protein had, on average, 2.1 interaction partners in the folded system, this number increased to 8.6 when the proteins are all unfolded (see Figure 2E). As a consequence, the average number of atom–atom contacts per protein rose by a factor of 25 upon unfolding. Among the different contact types, this increase was the strongest for “hydrophobic” contacts (i.e., between nonpolar atoms), which were enhanced by a factor of 41 (see Figure 2F). Recently, the diffusion slowdown in crowded solutions of globular proteins at intermediate concentrations (≤200 g/L) has been linked to the formation of transient protein clusters.^34,41^ Indeed, we observed that in folded systems with protein concentrations below 200 g/L, the proteins were organized in 1–3 clusters, with a few remaining protein molecules floating freely in solution (see Figure S13 in the SI). At higher concentrations, most of the time the folded proteins formed a single large cluster, while maintaining a degree of dynamical exchange with the bulk. On the contrary, in the case of the unfolded systems, we observe the formation of a single cluster encompassing all proteins and creating a stable network, regardless of the concentration. The enhanced stickiness of unfolded proteins was reflected by a strong rise in the solution viscosity. For a 288 g/L protein system at *T* = 300 K, the viscosity increased upon unfolding from η = 8 ± 1 to 60 ± 8 mPa·s with a99SB-disp and from η = 11 ± 3 mPa·s to as much as 360 ± 70 mPa·s with CHARMM36m (Figure S18). The substantial relative increase in viscosity caused by unfolding persisted even at the highest temperatures, (with η going up from 3.4 ± 0.8 to 18 ± 4 mPa·s for a99SB-disp and from 3.7 ± 0.3 to 72 ± 19 mPa·s for CHARMM36m at *T* = 380 K). Our results are consistent with the trend observed in previous experimental studies,^42−44^ reporting an increase in the viscosity of protein solutions undergoing thermal denaturation. Actually, owing to the significantly lower protein concentrations (below 100 g/L) that were examined in those measurements, the unfolded protein viscosities (∼1–40 mPa·s) are lower than our computational estimates. On the other hand, the viscosities of our unfolded systems are smaller than those reported for concentrated antibody solutions,^45^ reaching up to 1700 mPa·s, and several phase-separated biomolecular condensates.^46^ In fact, given the very slow decay of the pressure autocorrelation function (Figure S20), the viscosity values that we obtained for the unfolded systems likely represent a lower bound for the actual viscosity.

### Even Partial Proteome Unfolding Causes Strong Diffusion Slowdown

**Figure 3:**
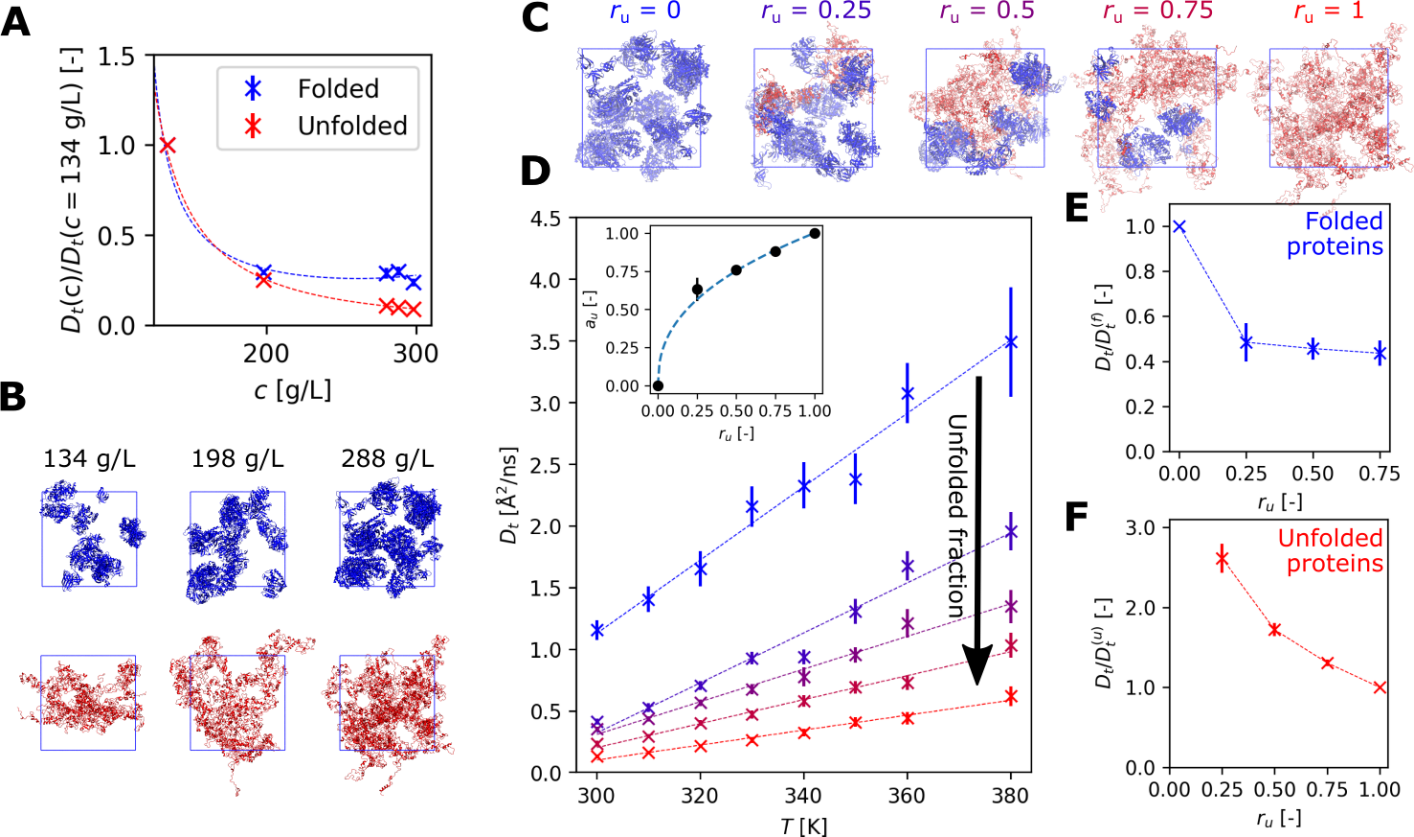
(A) Concentration-dependent diffusion slowdown from simulations. The slowdown is expressed relative to the diffusion coefficient for the least concentrated sub-box (134 g/L). The dashed lines represent fits with a second-order polynomial fraction. (B) Snapshots showing folded (upper row) and unfolded (lower row) simulation boxes at different protein concentrations. (C) Snapshots of sub-boxes (288 g/L) with a progressively increasing unfolded fraction *r*_u_. Folded proteins are blue while unfolded proteins are shown in red. (D) Translational diffusion coefficients in sub-boxes (288 g/L; see panel C) with a varying fraction of unfolded proteins. The insets show the dependence, fitted to a power law, of the apparent unfolded fraction *a*_u_ on *r*_u_. (E, F) The decrease in the translational diffusion coefficient of folded and unfolded proteins inside the partially unfolded sub-box (288 g/L) with increasing *r*_u_. The values shown in panels A, E, and F as well as in the inset of panel D are averages across all temperatures, with error bars expressing the standard deviations. The results presented in this figure were obtained with the a99SB-disp force field; analogous plots for CHARMM36m, exhibiting qualitatively the same behavior, can be found in the SI.

As expected,^20^ protein diffusion strongly decreases as a function of concentration (Figure 3A), with the sharpest drop occurring below 200 g/L. This effect is further accentuated for the completely unfolded systems. However, near the cell-death temperature only a fraction of the proteome might be unfolded.^11,13^ To explore the effect of such partial unfolding on the overall protein diffusivity, we performed additional temperature scans for a selected atomistic system (288 g/L, see also Figure 2B) with a varying fraction *r*_u_ of unfolded proteins (25%, 50%, and 75%; see Figure 3C). In each case, we completely unfolded the chosen amount of proteins while leaving the remainder fully folded. We selected the proteins to be unfolded randomly, trying to maximize the species’ heterogeneity in the chosen subset. Our simulations revealed that with the increasing unfolded content, the overall translational diffusion coefficients quickly approached those calculated for the fully unfolded system (see Figure 3D where we report data for the a99SB-disp force field). This effect is even stronger when using the CHARMM36m force field (Figure S11 in the SI). This finding demonstrates that the translational diffusion coefficient is a nonlinear function of the fraction of unfolded proteins, and it experiences a sharp drop already for small values of *r*_u_. To explain why *D*_t_ shows such a rapid drop, we separately analyzed the diffusion coefficients of folded and unfolded proteins in the intermediate boxes. We found that the *D*_t_ of folded proteins decreased by more than 50% already for the smallest *r*_u_ (see Figure 3E). Thus, the presence of even a small amount of unfolded proteins is able to strongly affect the diffusion of the remaining folded proteins. On the contrary, the diffusion coefficient of the unfolded proteins shows a more gradual decrease (Figure 3F). From a quantitative point of view, we express the translational diffusion coefficient in the partially unfolded proteome as , where *a*_u_ is an “apparent” unfolded fraction, weighting the diffusion coefficient of the fully folded and the fully unfolded systems,  and , respectively. It results that *a*_u_ shows a nonlinear dependence on the actual protein unfolded fraction *r*_u_ (see SI for a detailed discussion). This dependence, by considering an average over *T* for *a*_u_, can be fitted with the power law  (see the insets in Figures 3D and S11C), with the exponent *p* equal to 0.411 for a99SB-disp and 0.142 for CHARMM36m.

### Estimation of the Folding State of Cytoplasm and Validation of the Results

By combining the QENS and MD simulation results, it is possible to estimate the proteome stability curve, i.e., the temperature dependence of the unfolded protein fraction in the *E. coli* cytoplasm. For this purpose, first we described the temperature dependence of the apparent diffusion coefficient measured from QENS experiments (Figure 1A) with the empirical relation , in analogy to previous work on protein crowded solutions.^18^ In the fit the *D*^(f/u)^s are modeled by a linear function of *T* (see SI for details), while  is the apparent fraction of unfolded proteins in the system, as defined in the previous section and now derived explicitly from QENS data.

**Figure 4:**
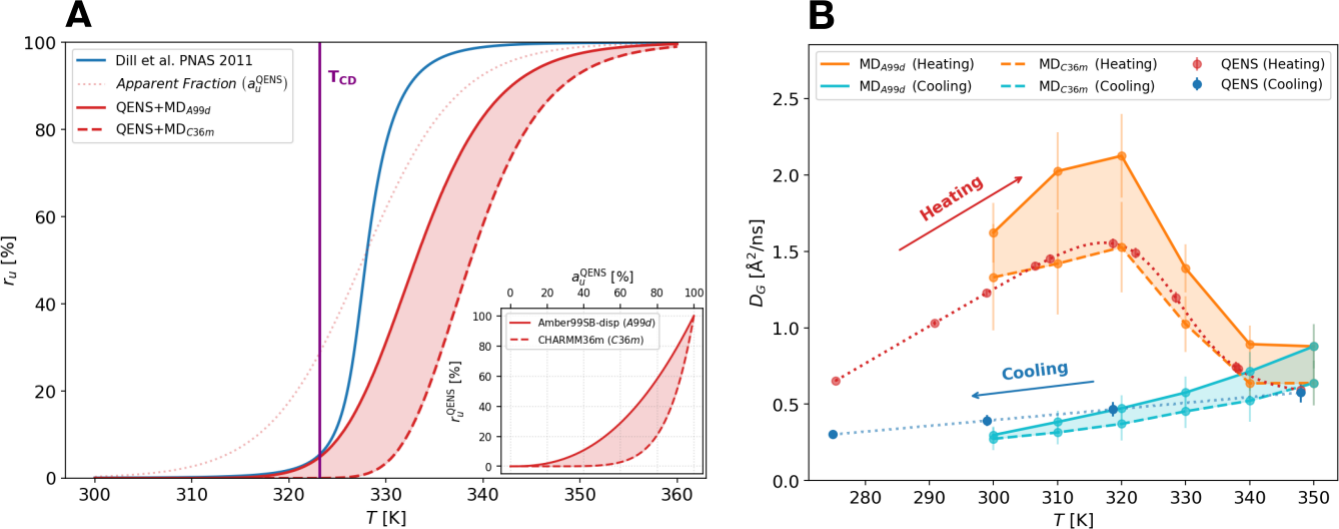
(A) Fraction *r*_u_ of unfolded proteins in the *E. coli* cytoplasm as a function of temperature. The cell-death temperature of 323.15 K,^10^ determined from the *E. coli* growth rate, is indicated by a vertical line. The red solid (a99SB-disp) and dashed lines (CHARMM36m) show  calculated by combining , extracted from the QENS measurement of the protein global diffusion with the force-field dependent relationship derived from simulations (see the inset in panel A). The results are compared with a theoretical prediction^10^ (blue solid line). (B) Comparison of the apparent global diffusion coefficient from QENS experiments (red and blue curves) with the apparent diffusion coefficient computed from simulations for the two force fields and by combining the contributions from translational and rotational motions (orange and cyan curves, see SI for details).

Second, we applied at each temperature the empirical power-law relationship between *r*_u_ and *a*_u_, which we obtained from the analysis of the protein translational diffusion in the molecular simulations, to the experimentally derived apparent fraction of unfolded proteins, . We obtained the experimentally derived fraction of unfolded proteins as a function of temperature, , see Figure 4A. This quantity is force field dependent via the exponent parameter *p*, and can now be compared with an equivalent function theoretically derived by Dill and co-workers,^10^ see Figure 4A.

Strikingly, at variance with the proteome catastrophe scenario,^10^ the present s show a very slow increase of the fraction of unfolded proteins as temperature crosses *T*_CD_ = 323.15 K. In contrast to the proteome catastrophe model, where at a few degrees above the cell death temperature more than 50% of the proteins undergo unfolding, our model predicts an unfolded fraction of less than 25% there (see also Table S9 in the SI). This result is in line with recent experiments on proteome thermal stability.^11,13^

The data analysis and the simulations required strong, albeit reasonable, assumptions (see SI). Apart from the hypothesis that we discussed above, that the main contribution to the QENS signal comes from the *E. coli* proteome,^27^ it is worth noting that in our simulations we represented a very simplified version of the *E. coli* cytoplasm, composed of just a small subset of proteins. What is remarkable, however, is that such a simplified representation is able to catch the main dynamic features of the system we investigated, possibly strengthening the picture we propose.

An additional support to our findings comes from the fact that the experimental apparent diffusion coefficient *D*_G_ of the average protein can be correctly described from the simulations just starting from the estimates of the apparent fraction of unfolded proteins and of  The curves are reported in Figure 4B, showing a very good agreement with the experimental data, thus endorsing the assumptions we made.

### Reproducing the Growth Rate of *E. coli*

**Figure 5:**
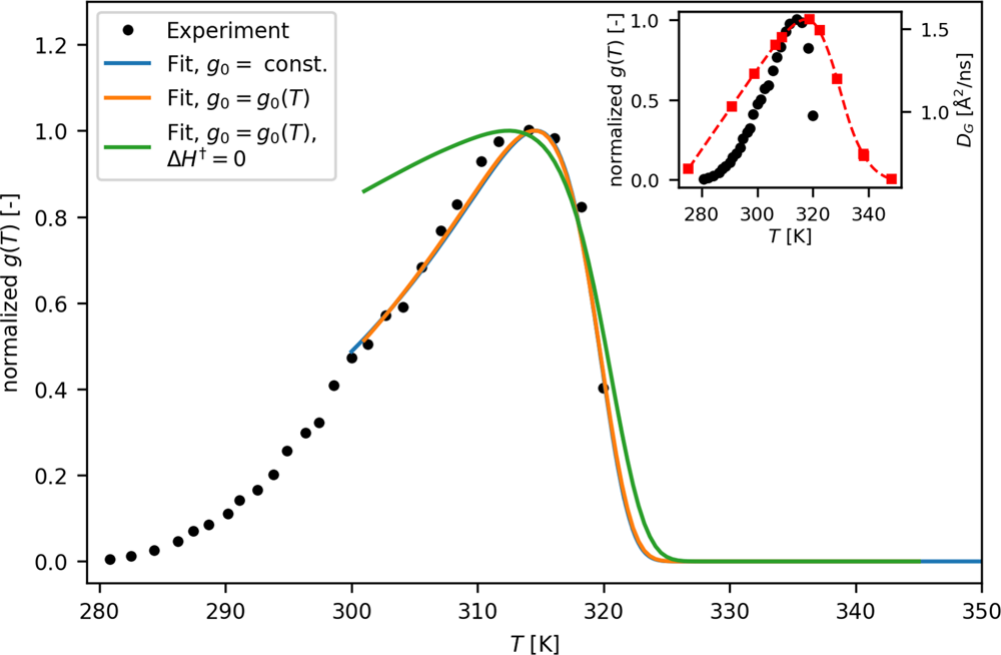
Growth rate of *E. coli* bacteria as a function of temperature. The fits of experimental data^47^ were obtained using a kinetic model for the growth rate based on the temperature-dependent fraction of unfolded proteins in the cytoplasm deduced by QENS/MD, , and the temperature-dependent diffusion constant measured by QENS. The fits were performed using a constant intrinsic growth-rate parameter *g*_0_ (blue line), a temperature-dependent  (orange) and assuming a negligible activation barrier Δ*H*^†^ (green). In the inset, the temperature dependence of the experimentally determined growth rate (black dots) is compared with that of  (red squares).

We used the stability curve of the proteome to reconstruct the growth-rate curve *g*(*T*) of *E. coli*. In the spirit of the approach described by Dill et al. in ref (10), *g*(*T*) is related to the temperature-dependent fraction of unfolded proteins via an Arrhenius reaction rate term, , where *g*_0_ is an intrinsic growth-rate parameter, Δ*H*^†^ is the dominant activation barrier, *RT* is the molar gas constant multiplied by temperature, Γ is the number of essential proteins for the bacterium growth, and *f*_*i*_ is the temperature-dependent fraction of folded proteins of species *i*. Using our results, we replace *f*_*i*_ with the average fraction of folded proteins estimated by QENS, , and we fit the experimental growth rate.^47^ As shown in Figure 5, when we use  from the a99SB-disp force-field, we obtain an excellent fit with the values Δ*H*^†^ ≃ 45 kJ/mol and Γ = 86. In the original model, and using a very different form of the stability curve, the values obtained were Δ*H*^†^ ≃ 27 kJ/mol and Γ = 51.^48^ We then introduce into the fit the temperature dependence of the exponential prefactor in terms of a reaction–diffusion model by assuming  (see Figure 1). The fit is comparable to the case where *g*_0_ is constant, but by including the dependence on diffusivity, we recover smaller activation barrier and number of essential proteins (Δ*H*^†^ ≃ 35 kJ/mol and Γ = 81). A final numerical test is done by assuming the growth rate of the bacterium completely rate-limited by diffusivity (Δ*H*^†^ = 0). In this case, the *E. coli* growth-rate curve cannot be fitted at temperatures below the cell-death. The obtained results confirm that even without assuming a proteome catastrophe, the *E. coli* growth rate can be reproduced very well by a smoother temperature progress of the proteome unfolding; and that the diffusion contribution to reactivity may help tuning the essential parameters of the model, i.e., Δ*H*^†^ and Γ.

## Discussion

We found that the thermal death of *E. coli* is signaled by a distinctive behavior of the proteome short-time dynamics: a strong decrease of the protein global diffusion coefficient starts just below *T*_CD_, from *D*_G_(320 K) = 1.5 Å^2^/ns down to *D*_G_(350 K) = 0.5 Å^2^/ns. The results from MD simulations show that this dynamic slowdown is due to the unfolding of a part of the proteome. This finding is consistent with previous experimental work where an analogous trend for the diffusive dynamics has been observed in concentrated protein solutions across the melting temperature.^17−19^ Therefore, protein unfolding dominates the observed temperature dependence, and in both cases the dynamic slowdown is irreversible.

Here, we take the analysis a crucial step forward by directly relating the diffusion coefficient to the amount of unfolded proteins in the system. The theoretical description of protein solutions containing a varying ratio of folded and unfolded proteins is challenging. These systems neither fit into the standard picture of solutions formed by globular proteins, usually modeled as rigid colloidal particles,^49^ nor can they be accurately described by means of simplified polymer models.^50^ In particular, limitations of the colloidal model were demonstrated for highly concentrated protein solutions involving changes in protein conformation,^51^ and the importance of protein–protein interactions was stressed.^52,53^

Our simulations showed that the presence of minor amounts of unfolded proteins causes a substantial slowdown in the protein global diffusion. As we demonstrated, this drop was not only due to the slower diffusion of the fraction of unfolded proteins, but also to a 2-fold slowdown of the remaining folded proteins as a consequence of the enhanced interactions with their unfolded counterparts. Thus, unfolded proteins, because of their extension and of the increased exposure of hydrophobic groups, form a sticky macromolecular network to which folded proteins associate. This resembles the behavior recently observed in biomolecular condensates, where the interactions of folded lysozyme proteins with a macromolecular network formed by pentameric constructs of SH3 domains, and containing disordered linkers, strongly affected the condensate viscoelastic properties.^46^

From the combination of QENS experiments and MD simulations, we estimated the amount of unfolded proteins in the cytoplasm at different temperatures. In the last years, there have been several attempts to connect the thermal death of bacteria to a critical amount of unfolding proteins. The aim was to understand if the death results from a collective unfolding of cell proteins^10,12^ or if it is caused by the denaturation of a subset of proteins controlling key biological functions.^11,13,54,55^ Here, we found that a few degrees above the cell-death temperature only a small fraction of proteins, less than 10%, are unfolded (see also Table S9 in the SI). This result supports the hypothesis first put forward by Leuenberger et al.^11^ that there is no catastrophic denaturation of the proteome, but instead only an unfolding of a subset of proteins.

It is important to stress that there is no unique definition of *T*_CD_ which, depending on the growth conditions of bacteria and their environment, can vary by several degrees Celsius. Owing to the predicted slow increase with temperature of the unfolded protein fraction (see Figure 4), this uncertainty does not affect our conclusions concerning the minor amount of unfolded proteins that are present in the cytoplasm at the cell death. Apart from the uncertainty in *T*_CD_, the quantitative estimate of the unfolded fraction may also be affected by some limitations of our computational model in terms of molecular composition, which focuses exclusively on proteins as the most prevalent type of macromolecules in the cytoplasm and which, moreover, is biased toward structurally well-resolved folded proteins. In addition, the model does not consider thermal adaptations of the proteome, such as evolving populations of heat-shock proteins and chaperons. However, even though the cells in the experimental samples still have a basal metabolism, they will have a reduced capability to tune the proteome composition due to the lack of nutrients. Finally, in our simulations we did not include metabolites, namely, the abundant ATP, which is essential for energy conversion and, by acting as a hydrotrope molecule, it may prevent protein aggregation in vivo.^56^ So far, its effect on protein (folded or unfolded) dynamics in crowded conditions has not been explored in detail. The modulation of protein diffusivity by ATP could affect the estimate of the fraction of unfolded proteins at the cell death.

The destabilization of the *E. coli* bacteria starts already at temperatures below the *T*_CD_. Temperatures near 315 K already represent a stress condition for the bacteria, they resist the increase in the environmental temperature with several active mechanisms, such as the change in the global protein population by increasing the number of molecular chaperones to maintain a properly folded proteome^57^ and the variation of internal viscosity by regulating the synthesis of glycogen and trehalose.^58^ Our experimental data shows that the proteome reaches a maximal diffusive mobility just in correspondence with the optimal growth temperature, above which a strong dynamical slowdown occurs (see the inset of Figure 5). This behavior is a consequence of the progressive unfolding of the bacterial proteome, which impacts in a different way on the decay of diffusive protein motions and the faster drop of the cellular growth rate. It is worth of note that just above *T*_CD_, the diffusion coefficient of the average protein decreases by a factor of 3 compared to the optimal growth temperature. In a simplified model, where *k*_on_ depends linearly on the protein diffusion coefficient,^16^ the rate of a reaction involving a pair of native proteins would consequently be reduced by the same factor, with the consequent vital impact on the related metabolic process.

In more detail, as our simulations have shown, a low amount of unfolded proteins can trigger an important slowdown of the diffusion of the surrounding macromolecules. The increase in local viscosity and the associated dramatically reduced protein diffusion caused by unfolding may threaten the viability of the cell by affecting localized physiological processes. A pertinent example at molecular scale is the dynamics and substrate channeling in enzyme assemblies,^59^ but also at larger scales the viscoelastic response of the cytoplasm associated with organelles’ localization.^60^ Moreover, we have shown that a very good reproduction of the *E. coli* growth rate can be obtained when combining together the fraction of unfolded proteins with the temperature-dependent diffusion within a simple reaction-diffusion model. This supports the idea that at least a part of the cell metabolism is modulated by diffusion. Finally, our findings are consistent with the idea that intrinsically disordered proteins may induce as well a mobility slowdown in the local environment, as it is probed in membrane-less organelles.^61^

The approach hereby presented can be extended, and possibly complemented by single-molecule techniques, to investigate the relationship between the dynamics and the proteome unfolding in extremophiles resisting either to cold or hot environments. Furthermore, attention could be enlarged to the peculiarity of the proteins’ dynamical response to stress in the functioning of specific networks of interaction, such as in the unfolding protein response cascade.

## Methods

### Sample Preparation

*E. coli* BL21 (DE3) was grown overnight in LB medium (made with H_2_O) at 37 °C with shaking (200 rpm). A total of 3.3 g of *E. coli* cells were collected by centrifugation and washed twice with a buffer made in D_2_O (99.9 atom D) as follows: cells were suspended in 36 mL of D_2_O buffer at pD8, spun at 5400*g* for 18 min at 6 °C and the supernatant was removed. The pD 8 buffer contained 50 mM Tris, 150 mM NaCl and 5 mM KCl. To obtain a pD of 8, the pH of the buffer was adjusted to 7.6 using HCl. The amount of measured *E. coli* sample was 600 mg. The dry-material content of the pellets was determined by freeze-drying as ≈20%.

### QENS Experiments

We performed two experiments^62,63^ on the cold neutron backscattering spectrometer IN16b at the Institut Laue-Langevin (ILL),^25^ with an energy resolution of ≈0.75 μeV fwhm, an energy range of |*E*| ≤ 31 μeV, and a wave-vector coverage of 0.19 Å^–1^ ≤ *q* ≤ 1.9 Å^–1^. The samples were measured for 2 h at each temperature in a temperature range from 275 to 348 K with uneven temperature steps. No unexpected or unusually high safety hazards were encountered. The data reduction (i.e., normalization to the monitor, integration over the regions of interest of the vertically position-sensitive detector tubes, calculation of the energy axis, and centering of the elastic line positions using separate Vanadium measurements) was carried out with the built-in module for IN16b of the Mantid program.^64^ The subtraction of the sample holder, the normalization to the detector efficiency, and the fit of the data were performed using an in-house python module. The fully reduced QENS data were described by the experimental scattering function *S*_exp_(*q*, *E*, *T*):
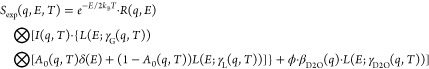
1where  is the detailed balance factor, *R*(*q*, *E*) is instrumental resolution, *I*(*q*, *T*) is the intensity of the signal due to the interaction of the neutrons with the average protein in the *E. coli* cytoplasm,^28^*L*(*E*; γ_G_(*q*, *T*)) and *L*(*E*; γ_L_(*q*, *T*)) are two Lorentzian functions accounting for the diffusive contributions deriving from the global and the local motions of the average protein, *A*_0_(*q*, *T*) represents the Elastic Incoherent Structure Factor (EISF) containing information on the geometry of the confined local motions, ϕ is a scalar factor that weights the contribution of the solvent,  is the amplitude of the solvent signal, and *L*(*E*; γ_D2O_(*q*, *T*)) is a Lorentzian function that takes into account the diffusive motions of the D_2_O molecules. Finally, the *q*-dependence of Lorentzian widths γ_G_(*q*, *T*) and γ_L_(*q*, *T*) has been described through the jump-diffusion model,^65^ as detailed in the SI. For further information on the analysis of the QENS data see the SI.

### MD Simulations

The LBMD simulations^36,66^ and the majority of the all-atom MD simulations were performed within the framework of the “Grands Challenges Joliot-Curie 2019” (GENCI) using the partition AMD Irene ROME of the TGCC Joliot Curie CEA HPC (25 million CPU hours). To effectively represent the protein composition of the *E. coli* cytoplasm, we created a 400 Å cubic box with 197 proteins of 35 different species selected on the basis of a previous computational model.^32^ The selection and modeling of the protein structures are detailed in SI. This large system was then equilibrated for 4.3 μs by performing an *NVT* LBMD simulation with the MUPHY software^67^ at *T* = 300 K. To explore the diffusion of proteins with the all-atom resolution, we extracted five representative cubic sub-boxes (170 Å) from different frames of the produced trajectory, and we converted them into the all-atom resolution (see SI for details). In addition, to investigate the effects of unfolding on the protein dynamics, we completely unfolded some or all protein structures in the sub-boxes through a set of all-atom MD simulations at high temperature (450–1500 K). With these folded as well as partially and fully unfolded atomistic subsystems, we performed a sequence of *NPT* MD simulations at increasing temperatures (102.4 ns per temperature step) to investigate the temperature dependence of the diffusion coefficients of the average protein in the subsystem and the effects of unfolding on the dynamics. All-atom MD simulations were performed using the GROMACS 2019.4 software^68^ and employing two distinct sets of force field parameters to describe the proteins: Amber a99SB-disp^39^ and CHARMM36m.^40^ The details of the LBMD and all-atom simulation protocols as well as those of the subsequent data analysis, including the calculation of diffusion coefficients and viscosities, are described in SI.

## Data Availability

The starting geometries used for the production simulations of the all-atom subvolumes together with the resulting mean squared displacement curves per individual protein/chain as well as the rotational autocorrelation functions per each chain are available at https://doi.org/10.5281/zenodo.7457333.

## Supporting Information Available

Additional details on neutron scattering experiments, molecular dynamics simulations, data analysis, and supplementary results (PDF)
