# Supplementary material for: Diffusive Dynamics of Bacterial Proteome as a Proxy of Cell Death
Source: ACS Cent Sci. 2023 Jan 9;9(1):93–102. doi: 10.1021/acscentsci.2c01078 (PMC9881203; doi:10.1021/acscentsci.2c01078)
Supplement: Supplementary file 1 — oc2c01078_si_001.pdf [file oc2c01078_si_001.pdf]

Supporting Information to: Diffusive Dynamics of Bacterial Proteome as a Proxy of Cell Death

Daniele Di Bari[a,b,c], Stepan Timr[d,e,f],
Marianne Guiral[g], Marie-Thérèse Giudici-Orticoni[g],
Tilo Seydel[c], Christian Beck,[c]
Caterina Petrillo[a], Philippe Derreumaux[d,e,j], Simone Melchionna[h,i],
Fabio Sterpone[d,e,*], Judith Peters[b,c,j,*], Alessandro Paciaroni[a,*]

[a]Dipartimento di Fisica e Geologia, Univesità degli Studi di Perugia
[b]Université Grenoble Alpes, CNRS, Laboratoire Interdisciplinaire de Physique
[c]Institut Laue-Langevin
[d]Laboratoire de Biochimie Théorique (UPR 9080), CNRS, Université de Paris
[e]Institut de Biologie Physico-Chimique, Fondation Edmond de Rothschild
[f]J. Heyrovský Institute of Physical Chemistry, Czech Academy of Sciences
[g]Laboratoire de Bioénergétique et Ingénierie des Protéines, IMM, CNRS, Aix-Marseille Université
[h]ISC-CNR, Dipartimento di Fisica, Università Sapienza
[i]Lexma Technology
[j]Institut Universitaire de France

Quasi-Elastic Neutron Scattering (QENS)

Model. The fully reduced experimental scattering function, $S_{exp}(q, E, T)$, can be obtained by the convolution of the theoretical scattering function S_{th}, describing the interaction between the neutrons and the bacteria, and the instrumental resolution, $R(q, E)$, which is determined by a vanadium sample, a completely elastic and incoherent scatterer (1):

$$S_{exp}(q, E, T) \;=\; e^{-\frac{E}{2k_{\mathrm{B}}T}} \cdot \left[R(q, E) \otimes S_{th}(q, E, T)\right], \tag{1}$$

where $e^{-\frac{E}{2k_{\mathrm{B}}T}}$ is the detailed balance factor.

Since proteins represent $\approx$ 55% of the bacterial dry weight and have a higher percentage of hydrogen content (50%) than any other type of macromolecules (30%) (2), the major contribution to S_{th} is originating from self-diffusive dynamics of an average protein and of the bulk water present in the sample (in this case D_2O):

$$S_{th}(q, E.T) \; = \; S_{AP}(q, E, T) + \phi \cdot S_{D2O}(q, E, T), \tag{2}$$

where $S_{AP}(q, E, T)$ and $S_{D2O}(q, E, T)$ are, respectively, the scattering functions of the average protein and the D_2O, and ϕ is a scalar factor that weights the contribution of the solvent. ϕ can be estimated by the product of the D_2O amount in the sample, which constitutes about 80% of the sample mass, and the percentage of bulk water molecules in *E. coli*, which is almost 60%.

Fitting procedure. To mitigate the problem of overfitting due to the complexity of the system and the consequent high number of unknown parameters necessary to describe the resulting QENS signal, we tried to reduce, as much as possible, the number of free parameters that can vary during the fit. To this end, we used the measurements of the PBS-D_2O buffer to fix the q-dependence of the solvent's intensity S_{D2O} in eq. (2). Moreover, we employed a three step procedure for the analysis, where we performed at each step a fit of the data and we used the resulting information to improve the model and to reduce the number of free parameters. An example of the results from this procedure is shown in Fig. S1A.

Energy resolution The instrumental resolution function $R(q, E)$ takes several parameters into account and its expression strongly depends on the set-up of the instrument. In particular, on the IN16b spectrometer the resolution function is well described by a sum weighted by the scalar parameter $F(q)$ of a Gaussian and a Lorentzian function (3):

$$R(q, E) = A(q) \cdot \left\{ F(q) \cdot \frac{1}{\pi} \cdot \frac{\gamma(q)}{\gamma^2(q) + [E - E_0(q)]^2} + (1 - F(q)) \cdot \frac{1}{\sigma(q)\sqrt{2\pi}} \cdot e^{-\frac{[E-E_0(q)]^2}{2\sigma^2(q)}} \right\} + B(q) \tag{3}$$

where $|F(q)| \leq 1$. B(q) reflects a background which can depend on q. Figure S1B shows the fit of the measured Vanadium data with the eq. (3) and the measured energy resolution of the instrument, ΔE_{FWHM}, corresponding to the *Full Width–Half Maximum* (FWHM) of eq. (3). The resulting parameters averaged over q are reported in table S1.

Solvent contribution. To simplify the fit of the *E. coli* data, we first analyzed the QENS data of the PBS-D_2O buffer at three temperatures: 276 K, 300 K, and 324 K to obtain an estimation of the q-dependence of the solvent intensity. The main contribution comes from D_2O bulk water whose scattering function can be well represented as follows (4):

$$S_{D2O}(q, E) \; = \; e^{-\frac{E}{2k_{\mathrm{B}}T}} \cdot R(q, E) \otimes \left[\beta_{D2O}(q) \cdot L_{\gamma_{D2O}}(E)\right], \tag{4}$$

where β_{D_2O} is the amplitude of the signal, and $L_{\gamma_{D2O}}$ is a Lorentzian function that describes the diffusive translational motions of the D_2O molecules. An example of the resulting parameters is reported in Fig. S2.

The D_2O cross section comprises 79% coherent and 21% incoherent scattering and, applying Vineyard's convolution approximation, we have $S_{D2O}(q, E = 0) \approx S_{D2O}^{(coh)}(q)$, where $S_{D2O}^{(coh)}$ is the coherent static structure factor (5). Hence, $S_{D2O}(q, E)$ has as main contribution the coherent scattering of D_2O (6). Note that the experimental intensity of $S_{D2O}(q, E)$ (Fig. S2) reproduces qualitatively well the shape of $S_{D2O}^{(coh)}(q)$ measured by Bosio et al. (7).

Average protein contribution. Protein dynamics at all the temperatures has been described in terms of two distinct diffusive processes arising from the dynamics of the average protein in the *E. coli* cytoplasm (See Fig. S1A), whose narrow and broad Half Width–Half Maximum (HWHM) of the signal γ_G and γ_L correspond, respectively, to the long and the fast characteristic timescales of the probed global (G) and local (L) motions. The dynamic scattering function of the proteins is modeled here by the following expression (2, 8):

$$S_{AP}(q, E, T) = I(q, T) \cdot \{L(E; \gamma_G(q, T)) \otimes [A_0(q, T)\, \delta(E) + (1 - A_0(q, T))\, L(E; \gamma_L(q, T))]\}\,, \quad (5)$$

where $I(q, T)$ is the intensity of the signal, which is related to small vibrations within the proteins, meanwhile $L(E; \gamma_G(q, T))$ and $L(E; \gamma_L(q, T))$ are two Lorentzian functions accounting for the diffusive contributions due to global motions (roto-translations of the entire proteins) and local dynamics (internal motions of sub-parts of the proteins, e.g. conformational changes) mentioned above. Finally, $A_0(q, T)$, which multiplies the delta function $\delta(E)$, represents the Elastic Incoherent Structure Factor (EISF) containing information on the geometry of the internal motions that are confined within the space-time window of the spectrometer (1).

Data analysis. As described in the previous paragraphs, we employed a three step procedure for the fit of the data.

In the 1st step, we performed a simple fit that treated the measured spectra independently, apart from the weighting factor of the solvent contribution ϕ, which was shared among the spectra since it should depend only on the amount of bulk D_2O in the samples. On the contrary, all the remaining parameters could vary both with q and T. This allowed us to study the trends of the Lorentzian widths $\gamma_i(q, T)$ as function of q^2, and we verified that, both for the global and the local motions, they are well described by a jump-diffusion model as already observed in previous *in vivo* QENS experiments on bacteria (2, 9, 10) (see Fig. S3 and S4):

$$\gamma_i(q, T) = \frac{q^2\, D_i(T)}{1 + q^2\, D_i(T)\, \tau_i(T)} \quad \text{for } i \in \{G, L\}. \quad (6)$$

Within this model, proposed by Singwi and Sjölander (11), the diffusion is assumed to occur via infinitely small, elementary jumps characterized by a negligible jump time and the residence time τ_i, i.e. the time a proton spends in a given position. This is consistent with results of previous *in vivo* QENS experiments on bacteria (2, 9, 10).

In the 2nd step, we performed a simultaneous fit of the spectra measured at different q-values assuming *a priori* the jump-diffusion model for both the global and the local dynamics, i.e. constraining the q-dependence of γ_G and γ_L by eq. (6). The resulting values of the diffusion coefficients and the residence times for the global and the local dynamics are reported in Fig. S5.

Around the cell-death temperature ($T_{CD} \approx 323.15K$), one sees an important slowdown of the entire protein described by D_G. It is possible to model the behavior by the function, which links the diffusion slowdown to the gelation of the system induced by protein unfolding (8, 12):

$$D_G(T) = (a_1\,T + b_1)\,\left[1 - a_u^{\text{QENS}}(T)\right] + (a_2\,T + b_2)\,a_u^{\text{QENS}}(T) \tag{7}$$

with:

$$a_u^{\text{QENS}}(T) = \frac{1}{1 + e^{-(T-T_0)/\Delta T}} \tag{8}$$

where a_u^{QENS} is a smeared step function describing the transition between the initial liquid state and the final gel-like state, that can be considered as a dynamical estimation of the apparent fraction of unfolded proteins in the *E. coli* cytoplasm T_0 corresponds to the temperature of the transition between the two states.

In the 3rd and last step, we constrained the temperature dependence of D_G by eq. (7), and we performed a simultaneous fit of all the measured spectra. The resulting parameters, which are constant in T and q, are reported in table S2. Note: Fig. 1D of the main text shows the $D_G(T)$ obtained with eq. (7) using the shared parameters reported in table S2.

An example of the resulting parameters for the EISF, $A_0(q)$, at five selected temperatures is shown in Fig. S6 (*left*). The EISF contains information on the geometry of localized motions within the protein. In particular, we found that the EISF can be well described by the model proposed by Grimaldo et al. in a previous study on γ-globulin (13):

$$A_0 = A_0(q,T) = p_L(T) + [1 - p_L(T)]\,[s_L(T)\,A_{\text{sph}}(q,T) + (1 - s_L(T))\,A_{\text{3JD}}(q,T)] \tag{9}$$

where:

$$A_{\text{sph}}(q,T) = \left(3 \cdot \frac{j_1(q \cdot r(T))}{q \cdot r_L(T)}\right)^2 \qquad \text{and} \qquad A_{\text{3JD}}(q,T) = \frac{1 + 2j_0(q \cdot a_M)}{3}. \tag{10}$$

p_L is the fraction of H-atoms appearing fixed on the accessible time scale of the instrument. For the remaining H-atoms, two types of motions were taken into consideration: confined

diffusion in an impermeable spherical volume of radius r_L, described by the amplitude A_{sph}, and random jump diffusion between three equidistant sites on a circle of radius a_M, arising from methyl-groups, and described by A_{3JD}, a_M being fixed to 1.715Å, which is the average distance of H-atoms in methyl groups. The relative contribution of these two diffusive processes is measured by s_L, which, more specifically, describes the fraction of H-atoms that, among those atoms that are not appearing fixed (i.e. $1 - p_L$), are undergoing the spherically confined diffusion. j_0 and j_1 are Bessel functions of the first kind.

The dashed lines in Fig. S6 (*left*) are the best fits of the EISF with (9). The parameters are reported in S6 (*right*). Comparing the fraction of H-atoms that are fixed with those that diffuse in a sphere or undergo a 3-jump diffusion (see Fig. S7), we find that the percentage of H-atoms that are diffusing in a spherical volume is constant in temperature. Therefore, the changes in the fraction of fixed atoms discussed in the first paragraph of the results section are due to the variation of the fraction of H-atoms undergoing the 3-jump diffusion, only.

Model preparation for simulations

Protein composition. To represent the protein composition of the *E. coli* cytoplasm, we built our simulation system on the basis of a previous computational model (14), which was derived from the results of a proteomics study (15) of *E. coli* grown under minimal media conditions. The computational model reported in (14) contained 45 different protein species as well as 5 types of RNA and RNA–protein complexes. For the purposes of the present study and in order to permit back-mapping of smaller sub-volumes into an all-atomistic resolution, we reduced the model in the following aspects. First of all, we considered a smaller cytoplasmic volume than (14), namely a 400 Å cubic box. Second, we focused exclusively on proteins since they are known to form the most abundant macromolecular type in the *E. coli* cytoplasm and since the second most abundant macromolecular type—the RNA—is mainly concentrated in large ribosomal particles, which we did not include in our model. Third, we only considered protein species with molecular weights below 150 kDa to avoid large protein oligomers that would not fit easily in back-mapped sub-volumes. We assumed the same target macromolecular concentration as (14), that is, 275 g/L. Analogously to (14), the number of copies of each individual protein species was based on the relative abundances reported in (15). From the list of proteins simulated by (14), we omitted those counting less than 0.5 copies per our simulation box. In addition, we did not include the GltD, Hns, Pnp, and Bcp proteins, each of which would only exist in one or two copies in our simulation box, owing to the absence of reliable structures for homology modeling. On the other hand, for computational investigations of protein unfolding that are not reported in this work, we included 10 small monomeric barrels of superoxide dismutase 1 (SOD1) (16), raising the total macromolecular concentration to 279 g/L. Overall, the system comprised 197 proteins of 35 species (see Table S3).

Obtaining protein structures. Where available, we used an *E. coli* PDB structure to prepare an atomistic model of each protein species. Unless we found a more recent and higher-quality structure in the PDB database (17), we selected the same PDB structure as was used in the previous work (14). In three cases, we made use of a homology model stored in the SWISS-MODEL repository (18) as a starting point of the model preparation. Where needed, we subsequently added missing residues and corrected non-proline cis peptide bonds using the MODELLER software, version 9.23 (19). The quality of the structure was checked with the MolProbity server (20). When present in the PDB structure, metal ions coordinated to the protein were included in the model; however, larger ligand molecules were omitted. With the exception of TufA and GpmA, the oligomerization state of the protein was taken to be the same as in (14). While TufA was modeled as a dimer by (14), the prediction of the PDBe PISA server (21) yielded an ambiguous result, and the protein was described as a monomer in a previous experimental work (22). Therefore, we modeled TufA in a monomeric state. Similarly, while the GpmA protein was simulated in a dimeric form by (14), the PDBe PISA prediction placed it in a grey zone, and according to the EcoCyc database (23), the ATP-free version of the protein was monomeric. As a result, we considered GpmA in a monomer state.

The atomistic protein structures were hydrogenated by the GROMACS `pdb2gmx` tool (24), placed in rectangular boxes with a minimum distance of 1.5 nm between the protein and the boundary of the box, and solvated in a 150 mM KCl solution, including several additional ions to neutralize the net charge of the system, if needed. Each system was then subjected to a short energy minimization and equilibration protocol, performed in the GROMACS 2018.7 software (24). First, an energy minimization was performed to bring the maximum force below 1000 kJ mol^{-1} nm^{-1}, while harmonic restraints were applied to all heavy atoms of the protein (with a force constant $k_{\mathrm{BB}} = 1000$ kJ mol^{-1} nm^{-2} assigned to the backbone atoms and a force constant $k_{\mathrm{SC}} = 500$ kJ mol^{-1} nm^{-2} applied to the side-chain atoms). This energy minimization was followed by a six-step relaxation protocol with gradually decreasing harmonic position restraints, where the first two short simulations were performed in the NVT ensemble and were followed by four NPT trajectories simulated at a pressure of 1.01 bar (see Table S5 for more details). The minimization and equilibration protocol was performed separately for the Amber a99SB-disp (25) and CHARMM36m (26) force fields. The same simulation parameters were used as for the respective a99SB-disp and CHARMM36m systems described in the subsection *All-atom simulations*, except for the temperature coupling, which was ensured by the Berendsen thermostat (27) with a time constant of 1 ps. The equilibrated protein structures obtained with the a99SB-disp force field were converted to the OPEP description by the OPEP File Generator (http://opep.galaxy.ibpc.fr).

Initial positions and orientations of the proteins in the simulation box were generated by the Packmol software, version 18.169 (28). To make the spatial distributions of different protein species more uniform, each protein entity was treated separately in the Packmol input.

LBMD simulation

We performed a coarse-grained simulation of the large system using the lattice Boltzmann molecular dynamics (LBMD) approach (29, 30), implemented in-house in the MUPHY software (31). Our LBMD scheme, which has been successfully applied to a number of biological systems (32–35), combines a coarse-grained protein model with a lattice-based description of hydrodynamic interactions (30). The protein model was based on the OPEP v.4 force field (36). Since the goal of this coarse-grained simulation was to sample different intermolecular arrangements rather than simulate protein conformational changes, the conformations of the proteins were restrained by an elastic network (distance cutoff 6 Å, force constant 5 kcal mol^{-1} $Å^{-2}$). The primary focus on intermolecular interactions also allowed us to simplify the description of the protein backbone, which was exclusively represented by Cα beads, the sizes of which were increased by 50 % relative to the standard OPEP v.4 model. Finally, the OPEP sidechain–sidechain non-bonded interactions were rescaled by a factor of 0.857 to mitigate excessive aggregation (37).

The simulation was performed in an NVT ensemble at $T = 300$ K, using a time step of 10 fs for bonded interactions and a time step of 20 fs for non-bonded and hydrodynamic interactions. A trajectory length of 4.3 μs was reached. Hydrodynamic interactions were described using the lattice Boltzmann (LB) technique (38), employing the BGK (Bhatnagar-Gross-Krook) collisional operator (39), with a lattice grid spacing of 4 Å. The LB kinematic viscosity ν_0 was set to reproduce bulk water behaviour at ambient conditions. The coupling coefficient γ was determined for each protein species as a function of its molecular weight (see Figure S14). The dependence of γ on molecular weight was derived by performing separate simulations of six protein species (namely CspC, SOD1, Adk, TufA, MetE, and GapA) of different molecular weights in dilute conditions and by adjusting the γ of each species to match the diffusion coefficient of each isolated species with a prediction obtained with the HYDROPRO software (40).

Sub-volume selection and back-mapping

To explore the diffusion of proteins with the all-atom resolution, allowing the description of conformational flexibility and unfolding, we selected five sub-volumes sampled in the LBMD trajectory and converted them into the all-atomistic resolution.

The selection of the sub-volumes proceeded in the following way. A cube with an edge length of 170 Å was placed in randomly chosen trajectory frames over the first 3 μs of the LBMD trajectory, with random positions and orientations (60,000 trials). In the dense environment of the cytoplasmic system, it was practically impossible to find sub-volume positions and orientations so as to avoid cutting at least a few proteins by the sub-volume boundary. For each placement, proteins that were fully contained in the sub-volume were counted as well as those that were cut by the sub-volume boundary. Subsequently, we isolated 120 sub-volumes that minimized the ratio of the total mass of proteins cut

by the sub-volume boundary versus the total mass of proteins entirely placed inside the sub-volume. If we had only kept those proteins that were not cut by the sub-volume boundary, we would have systematically underestimated the protein concentration in the sub-volumes. Therefore, for each of the 120 sub-volumes, the proteins that were integrally inside the sub-volume were complemented by a subset of proteins that were cut by the boundary. This subset was constructed in the following way. First of all, we only retained proteins the periodic images of which did not overlap with any protein that was integrally placed inside the sub-volume. An overlap was defined as a close contact between two protein beads, with a distance less than 3 Å. Next, we checked for overlaps among the proteins that we retained in the previous step, and we removed, one by one, proteins that exhibited the largest number of overlaps with the remaining members of the subset. In this way, we ended up with a subset of proteins that had no overlap with any other protein in the sub-volume.

Out of the 120 sub-volumes constructed with the procedure described above, we selected a representative set of five sub-volumes that 1) approximated the distribution of local protein concentrations inside the large volume and that 2) maximized the diversity of protein species represented in the set while approximating the number distribution of protein species in the large volume. The distribution of local protein concentrations (see Figure 2B) was obtained by randomly placing a 170 Å cube over the trajectory (2000 trials) and by evaluating the protein concentration inside the cube for each placement.

The protein concentration and species composition of each of the five cubic sub-volumes, sampling the structure and local concentration of the crowded solution, is indicated in Table S4.

Each sub-box, with a size of 170 Å and containing between 11 and 20 proteins, was subsequently converted into the all-atom resolution using the following back-mapping protocol. Atomistic structures prepared as described in the paragraph *Obtaining protein structures* were overlapped with the geometries from LBMD by aligning the Cα atoms of the atomistic structures with the corresponding Cα beads. We checked the back-mapped configuration for the presence of entangled aromatic side chains and corrected this artifact if needed. The boxes were subsequently hydrated, and a 150 mM concentration of K^+ and Cl^- ions was added, including extra ions neutralizing the net charge of the proteins. The systems were then energy-minimized and equilibrated using a protocol described below.

All-atom simulations

Force field and simulation parameters. All-atom molecular dynamics (MD) simulations were performed using the GROMACS 2019.4 software (24). We employed two distinct sets of force field parameters to describe the proteins: Amber a99SB-disp (25) and CHARMM36m (26), which belong to the most recent generation of protein force fields and which were optimized to correctly capture the properties of unfolded ensembles. The a99SB-disp protein force field was coupled with the a99SB-disp water model,

while CHARMM36m used the TIP3P water model (41). In both cases, K^+ and Cl^- ions were described with the default parameters for the respective force field. A 1.2 nm cutoff was applied to short-range non-bonded interactions. In addition, van der Waals forces were smoothly switched to zero between 1.0 and 1.2 nm in the CHARMM36m simulations. Long-range electrostatic interactions were evaluated using the particle mesh Ewald method (42). Newton's equations of motion were propagated using the leap-frog algorithm (43). The LINCS algorithm (44) was used to constrain the lengths of all protein bonds involving hydrogen, and the SETTLE algorithm (45) was employed to keep water molecules rigid.

The temperature of the system was maintained by the velocity rescaling thermostat with a stochastic term (46), which was coupled separately to the proteins and to the rest of the system with a time constant of 0.1 ps. The pressure was kept at 1.01 bar by the Parrinello-Rahman barostat (47) in all the production runs and by the Berendsen barostat (27) in all the NPT equilibration steps, with the time constant equaling 2 ps for both barostats.

Sub-volume equilibration. The procedure that we employed to equilibrate the atomistic sub-volumes at different temperatures and both in folded or unfolded states is detailed in Figure S15). First, the configurations back-mapped from LBMD underwent a short initial relaxation (Figure S15B). Subsequently, a heating protocol (Figure S15C) was used to equilibrate folded sub-volumes at increased temperatures, ranging between 310 and 380 K. In parallel with this, an unfolding protocol (Figure S15D) and a subsequent cooling protocol (Figure S15E) were employed to produce unfolded sub-volumes and equilibrate them at different temperatures in the range between 300 and 380 K. To efficiently heat up or cool down the system in the heating and cooling protocols, a heating sequence was used (48), enabling us to quickly reach the desired temperature while allowing the system to equilibrate. To follow the unfolding of the proteins during the unfolding protocol, we measured the root-mean-square deviation (RMSD) of each protein's atoms, its radius of gyration (R_g), as well as the amount of secondary structure present in the system, as determined by the DSSP software (49, 50) (Figure S10).

Preparation of partially unfolded sub-volumes. The partially unfolded sub-volumes were derived from the same initial configuration as the 288 g/L sub-volume (Table S4). The proteins selected for unfolding are listed in Table S6. The unfolded fraction r_u was quantified by computing the fraction of hydrogen atoms belonging to the unfolded protein structures. The sub-volume preparation followed a protocol equivalent to that employed to obtain fully unfolded sub-volumes (Figure S15). In addition, the positions of the atoms in proteins deemed to remain folded were kept frozen during the unfolding protocol (Figure S15D) as well as during the three pre-cooling stages of the cooling protocol (Figure S15E). Moreover, before the subsequent 100 ns NPT equilibration at 360 K, the frozen folded structures were gradually relaxed using the same six-step NVT equilibration as in

the initial relaxation protocol (Figure S15B); however, in contrast to the initial relaxation, the decreasing harmonic position restraints acted on the atoms of the folded structures only.

Production simulations. The production runs following the equilibration procedure were performed at eight different temperatures (300, 310, 320, 330, 340, 350, 360, and 380 K) both for folded and unfolded sub-volumes with a time step of 2 fs and each reaching a trajectory length of 102.4 ns. The translational velocity of the center of mass of the whole system was removed every 100 steps. For the 288 g/L sub-volume simulated using the CHARMM36m force field, the production trajectories performed at $T = 330$ K were extended to 1 µs for further analysis (see Figure S12).

Calculation of D_t and D_r

Obtaining average D_t per sub-volume. To evaluate the average protein translational diffusion coefficients D_t for a given sub-volume, folding state, and temperature, the production trajectory was divided into 20 ns blocks. In each block, we first calculated the mean squared displacement (MSD) of the center of mass of each protein molecule. We then computed the average of these MSD curves weighted by the numbers of atoms of the proteins (see Figure S16). This average MSD curve was subsequently fitted with a straight line in the 0.3–5 ns regime, and D_t was extracted from the slope of the fit. Subsequently, we determined the average of D_t over the 20 ns blocks as well as the corresponding standard error of the mean. For the given sub-volume, folding state, and temperature, the resulting D_t was corrected for the effects of periodic boundary conditions (PBC) (see the Subsection *Correcting D_t and D_r for PBC effects*). The estimated error of the PBC correction was then added to the standard error of the mean determined from the block averaging to express the uncertainty of D_t. To obtain a final value of D_t characterizing the entire cytoplasm, we calculated an average of the PBC-corrected D_t's over all the sub-volumes, with the individual D_t's being weighted by the numbers of protein hydrogen atoms in the sub-volumes. An analogous weighted average of the error bars was performed to estimate the uncertainty of the final value of D_t.

Calculating D_t and D_r for the evaluation of D_G. To compute the apparent diffusion coefficient D_G, a quantity directly comparable with the experimental observable (see the Subsection *Evaluation of D_G*), it was necessary to calculate D_t and the rotational diffusion coefficient D_r separately for each individual protein chain. The reason for considering individual chains rather than entire protein molecules lies in the fact that upon unfolding, the different subunits of a protein may dissociate, and as a consequence, the evaluation of D_r for the entire molecule would no longer be meaningful.

For each protein chain, D_r was computed by using the following approach (51, 52), approximating the chain rotation as being isotropic. One thousand randomly distributed

unit vectors were centered and rotated along with the protein chain. This was done—for each frame—by fitting the geometry of the protein chain with a reference structure to which the fixed orientations of the unit vectors had been set. An autocorrelation function of the unit vectors' directions was then calculated by employing the `gmx rotacf` tool (24) and using the second-order Legendre polynomial for evaluating the autocorrelation. The resulting autocorrelation curve was fitted with an exponential function in the 0.3–5 ns regime to obtain the decay time τ which was subsequently used for calculating D_r as $D_r = 1/(6\tau)$.

To achieve the best possible convergence, the single-chain D_t and D_r were calculated from the entire trajectory without cutting it into multiple blocks.

Viscosity calculations

We estimated the low-frequency, low-shear viscosity η of the 288 g/L sub-volume (see Table S4) at three different temperatures (300 K, 320 K, and 380 K) for both the folded- and the unfolded systems. We also considered the intermediate unfolded systems with 25 %, 50 %, and 75 % of unfolded proteins (Table S6) at T = 300 K. All the calculations were repeated for both sets of force field parameters employed in this study.

To calculate η, we followed a similar approach as was used in (53). For each system and temperature, we extracted 30 snapshots (50 for the unfolded CHARMM36m system at 300 K) in 1 ns intervals from the final part of the respective production run. Each of those snapshots served as a starting point of a 1 ns NPT equilibration followed by a 10–30 ns NVT simulation (10 ns for the folded systems, 20 ns for the rest of the a99SB-disp systems, and 30 ns for the remaining CHARMM36m systems). The NVT simulation was used to sample the elements of the pressure tensor, which was saved in 10 fs intervals. The viscosity was calculated from an integral of the autocorrelation functions of the pressure tensor fluctuations. Namely, for each off-diagonal element of the pressure tensor ($P_{ij} = P_{xy}$, P_{xz}, and P_{yz}) and for each of the three combinations $P_{ij} = (P_{xx} - P_{yy})/2$, $(P_{xx} - P_{zz})/2$, and $(P_{yy} - P_{zz})/2$ of its diagonal elements, we computed the running integral

$$\eta_{ij}(t) = \frac{V}{k_B T} \int_0^t \langle P_{ij}(0) P_{ij}(\tau) \rangle \, \mathrm{d}\tau \tag{11}$$

where V represents the volume of the simulation box. For each ij, the mean of $\eta_{ij}(t)$ over all the NVT simulations was fitted with an analytical function corresponding to a bi-exponential decay of the autocorrelation function:

$$\eta_{ij}(t) = A\,\alpha\,\tau_1(1 - e^{-t/\tau_1}) + B\,(1 - \alpha)\,\tau_2(1 - e^{-t/\tau_2}) \tag{12}$$

where A, B, α, τ_1, and τ_2 were parameters of the fit. The cutoff time t_{cut} for the fitting was determined as the time where the standard error of the mean of $\eta_{ij}(t)$ for the given ij exceeded 20 % of the mean. Examples of $\eta_{ij}(t)$ and the fits can be found in Figure

S20. The η_{ij} was then calculated as the limit of $\eta_{ij}(t)$ at infinity. Finally, the value of η was obtained as the mean of the six η_{ij} values, and the uncertainty of η was determined as the standard error of the mean of the six η_{ij} values.

To obtain a dilute reference, we also calculated the viscosity of an aqueous solution of 150 mM KCl considering the two respective water models—the a99SB-disp water model and the TIP3P model—and the same three temperatures as above. In each case, we performed a 100 ns NVT simulation of a 5.1 nm cubic box after a short energy minimization and a 1 ns NPT equilibration. We split the simulation into 10 ns blocks and computed the autocorrelation functions $\langle P_{ij}(0)P_{ij}(t)\rangle$ separately for each block. Next, making use of the isotropy of the KCl solution, we averaged the autocorrelation functions over ij for each block and calculated the running integral $\eta(t)$ from the average. Subsequently, the average of $\eta(t)$ from all the blocks was fitted—using a 10 ps cutoff—with the same analytical function as shown in eq. (12). Analogously to the case of the crowded sub-volumes, η was computed as the limit of the analytical function at infinity.

To estimate the correction to the diffusion coefficients due to periodic boundary conditions, we interpolated the viscosity to intermediate temperatures and extrapolated it to different protein concentrations by using analytical expressions derived in (54). First, we interpolated the temperature dependence of the solvent viscosity $\eta_0(T)$ using the following expression (54):

$$\eta_0(T) = \exp\left(-B_\mathrm{w} + D_\mathrm{w}T + \frac{\Delta E_\mathrm{w}}{RT}\right) \tag{13}$$

The curves resulting from these interpolations are shown in Figure S17, and the corresponding values of the parameters B_w, D_w, and ΔE_w are listed in Table S7. As a next step, we interpolated the viscosities of the crowded system to different temperatures using the relationship (54):

$$\eta(T, c) = \eta_0(T)\,\exp\left[\frac{c}{\alpha - \beta c}\left(-B + DT + \frac{\Delta E}{RT}\right)\right] \tag{14}$$

In this expression, c denotes the protein concentration while B, D, and ΔE are free parameters. The parameter α is a function of the protein molecular weight M_p, the molecular weight of water M_w, and its density ρ_w:

$$\alpha = \rho_\mathrm{w}\frac{M_\mathrm{p}}{M_\mathrm{w}} \tag{15}$$

We set M_p to 54.66 kDa, i.e., to the average molecular weight of the proteins in our model. The parameter β was defined as:

$$\beta = \alpha\nu - 1 \tag{16}$$

where we set ν to a value that was determined previously for BSA, ν = 1.417 x 10^{-3} m^3/kg (54) The parameters B, D, and ΔE obtained for each force field and for both folded and unfolded systems are listed in Table S8, and the resulting $\eta(T, c)$ functions for

$c = 288$ g/L are displayed in Figure S18A–B. As illustrated in Figure S18C, the analytical function $\eta(T, c)$ defined by eq. (14) allowed us to extrapolate the dependence of viscosity on concentration for a given T.

To estimate the uncertainty of the interpolated/extrapolated viscosity values, we repeated the fitting of eq. (14) with random combinations of viscosities each sampled within the confidence interval at the given temperature.

Correcting D_t and D_r for PBC effects

To correct the translational diffusion coefficients for PBC effects, we added the following term (55) to D_t computed from simulations:

$$D_t^{\mathrm{corr}} = \frac{k_{\mathrm{B}} T \xi}{6\pi\eta(T, c)L} \tag{17}$$

where $\xi = 2.837297$, L is the side length of the cubic box, and $\eta(T, c)$ is the function defined in eq. (14) and parameterized on the basis of viscosity values computed from simulations (see the Subsection *Viscosity calculations*). The uncertainty in the value of $\eta(T, c)$ was propagated to estimate the error of the correction term D_t^{corr}.

Similarly, we computed the correction to the rotational diffusion coefficient as (56)

$$D_r^{\mathrm{corr}} = \frac{k_{\mathrm{B}} T}{6\eta(T, c)L^3} \tag{18}$$

Evaluation of D_G

In order to directly compare simulations with experiments, we estimated each protein's apparent diffusion coefficient D_G, which takes into account not only the translational motions, but also the rotations of the entire protein. To this end, we used the following relation that links D_G to D_t and D_r (4, 57):

$$\sum_{n=0}^{N} B_n(q) \cdot \frac{D_r\, n(n+1) + (D_t - D_G)\, q^2}{\left[D_r\, n(n+1) + (D_t + D_G)\, q^2\right]^2} \xrightarrow[N\to\infty]{} 0 \tag{19}$$

where $B_n(q)$ is:

$$B_n(q) = (2n+1) \int_0^{\infty} \rho_H(r) \cdot j_n^2(qr)\, dr \tag{20}$$

and j_n is the spherical Bessel function of order n and ρ_H is the radial distribution function (RDF) of the protein hydrogen atoms.

After the calculation of D_t and D_r for each protein (as described in the previous section), we fixed $N = 550$ (4) and we solved numerically eq. (19) for three values of q: 0.2 Å^{-1}, 1 Å^{-1}, and 2 Å^{-1}, verifying that, in the q-range explored by the experiment

(0.2 Å$^{-1} \leq q \leq 2$ Å^{-1}), the value of D_G is constant. Indeed, if N and q are big enough, it is possible to show that the results do not depend on those parameters (4).

In this way, we obtained the D_G of each protein in the folded and the unfolded configuration at all the temperatures. Then, aiming to compare these data with the QENS results, for each system we calculated the average value of D_G by weighting each protein's contribution with the number of its hydrogen atoms. Figure S22 shows the results for the five selected sub-volumes extracted from the larger cubic box used for the LBMD simulations.

To obtain the value of D_G at intermediate folding states of the cytoplasm, we used the following relation:

$$D_G(T) = D^{(f)}(T) \cdot [1 - a_u^{\mathrm{QENS}}(T)] + D^{(u)}(T) \cdot a_u^{\mathrm{QENS}} \tag{21}$$

For further details on the validity of this approach, see the following subsection "*Connection between the apparent and the real unfolded fractions*". The resulting values of D_G, for CHARMM36m, are reported in Figure S23.

Finally, in order to calculate the most representative value of D_G for the average protein in the *E. coli* cytoplasm, we considered the probability distribution $p(c)$ for the selection of a sub-volume of $(17\ \mathrm{nm})^3$ and concentration c from the larger system with a volume of $(40\ \mathrm{nm})^3$ used in the LBMD simulation as representative of the bacterial cytoplasm; see Figure S24A. The weight of each sub-volume (Figure S24B) was calculated as follows:

$$w(c_i) = \frac{p(c_i)}{\sum_{k=1}^{5} p(c_k)} \tag{22}$$

The results, obtained with both force fields, are shown in Figure 4B.

Connection between the apparent and the real unfolded fractions

As we detailed in the subsection "*Neutron scattering data analysis*", we found that D_G measured by QENS experiments can be described as:

$$D_G(T) = (T \cdot a_1 + b_1) \cdot [1 - a_u^{\mathrm{QENS}}(T)] + (T \cdot a_2 + b_2) \cdot a_u^{\mathrm{QENS}}(T) \tag{23}$$

where $a_u^{\mathrm{QENS}}(T)$ is a smeared step function parameterized by the temperature of transition T_0 and the width of the transition ΔT:

$$a_u^{\mathrm{QENS}}(T) = \frac{1}{1 + e^{-\frac{T - T_0}{\Delta T}}} \tag{24}$$

The function $a_u^{\mathrm{QENS}}(T)$ weights the transition between two different dynamical regimes characterized by the two extremal diffusion coefficients $D_1(T)$ and $D_2(T)$, and since $D_2(T) < D_1(T)$, $a_u^{\mathrm{QENS}}(T)$ is a measure of the diffusion slowdown. At low temperatures

$(T \ll T_0)$, the parameter a_u^{QENS} tends to zero; therefore, $D_G(T) \approx D_1(T) = T \cdot a_1 + b_1$. At high temperatures $(T \gg T_0)$, the smeared step function is close to one, and $D_G(T) \approx D_2(T) = T \cdot a_2 + b_2$. However, the observed dynamical transitions is not reversible—after the transition, $D_G(T)$ remains equal to $D_2(T)$, even at low temperatures. The same dynamical behavior was previously found for proteins in solutions and, due to the similarity of the systems, this suggests that the underlying process is the same. In particular, in the first state (state 1), the *E. coli* cytoplasm is a liquid where the majority of proteins are folded. After the transition (state 2), the cytoplasm is in a gel state, and the proteins are mainly unfolded. For simplicity, we will assume that in states 1 and 2, all the proteins are fully folded and fully unfolded, respectively. Therefore, the diffusion coefficient D_1 will describe the global diffusion of the *folded* average protein in the liquid state, termed $D^{(f)}$, whereas D_2 will be related to the global diffusion of the fully *unfolded* average protein in the gel state, termed $D^{(u)}$. Thus, for $D_G(T)$ we have:

$$D_G(T) = D^{(f)}(T) \cdot [1 - a_u^{\mathrm{QENS}}(T)] + D^{(u)}(T) \cdot a_u^{\mathrm{QENS}}(T) \tag{25}$$

In this picture, $a_u^{\mathrm{QENS}}(T)$ can be interpreted not only as a measure of the diffusion slowdown during the unfolding, but it also represents the apparent fraction of unfolded proteins that weights $D^{(f)}$ and $D^{(u)}$ and that is necessary to reproduce the values of D_G for systems in a partially unfolded state. In particular, our simulations suggested that the relation between the apparent fraction of unfolded proteins determined dynamically, a_u^{MD}, and the real fraction of unfolded proteins, r_u^{MD}, can be described by a power law:

$$a_u^{\mathrm{MD}} = \left[r_u^{\mathrm{MD}} \right]^p \tag{26}$$

Therefore, to estimate the real unfolded fraction as a function of temperature starting from the dynamical QENS results, we can use the inverse relation:

$$r_u(T) = \left[a_u^{\mathrm{QENS}}(T) \right]^{(1/p)} \tag{27}$$

In conclusion, comparing QENS experiments and MD simulation, first we verified that the simulations can reproduce the linearity of $D^{(f)}(T) = D_1(T) = T \cdot a_1 + b_1$, and $D^{(u)}(T) = D_2(T) = T \cdot a_2 + b_2$ (Figure 2D and S11A). Then, since protein unfolding is computationally demanding for all-atom MD simulations, instead of calculating extremely long trajectories to allow all proteins to unfold, we forced the unfolding (as described in Subsection "*Preparation of partially unfolded sub-volumes*"), and we performed a series of relatively short simulations (∼100 ns), sufficiently long to determine the diffusion coefficient in the timescale explored by the QENS experiments, but brief enough to ensure that the folding state of the proteins was not affected. This allowed us to verify that if the folding state remains constant, the diffusion coefficient of the average protein scales linearly with the temperature (Figure 3D and Figure S11 panel C), and this relation also holds for systems with different concentrations (Figure S22).

Comparing the diffusion coefficients calculated for a system simulated in different folding states (Figure 3D and S11C), we found a relationship between the amount of unfolded proteins and the consequent diffusion slowdown—eq. (26). Finally, with the inverse relation (eq. (27)) and the QENS measurement of the slowdown, we were able to estimate the real fraction of unfolded proteins at different temperatures in the *E. coli* cytoplasm through the study of its dynamical state (see Figure 4A).

Supplementary Results

Origin of the nonlinearity of the transfer function

The observation that the translation diffusion coefficient D_t cannot be expressed simply as

$$D_t(r_u) = (1 - r_u)D_t^{(f)} + r_u D_t^{(u)} \tag{28}$$

where r_u is the unfolded fraction and $D_t^{(f)}$ and $D_t^{(u)}$ are the translational diffusion coefficients corresponding to a fully folded- and a fully unfolded system, respectively, can be rationalized in the following way. If we assume the validity of the Stokes–Einstein relation, the diffusion coefficients $D_t^{(f)}$ and $D_t^{(u)}$, forming two limiting cases, can be written as

$$D_t^{(f)} = \frac{k_\mathrm{B}T}{6\pi\eta^{(f)}R^{(f)}} \tag{29}$$

and

$$D_t^{(u)} = \frac{k_\mathrm{B}T}{6\pi\eta^{(u)}R^{(u)}} \tag{30}$$

Here, $R^{(f)}$ and $R^{(u)}$ are protein hydrodynamic radii in the folded and unfolded states, respectively, and $\eta^{(f)}$ and $\eta^{(u)}$ denote the viscosities of the fully folded and fully unfolded volumes.

However, in intermediate boxes with an unfolded fraction r_u, $\eta(r_u)$ is neither identical to $\eta^{(f)}$ nor to $\eta^{(u)}$. Consequently, for the diffusion of folded/unfolded proteins in the intermediate boxes, we can expect

$$D_t^{(f)}(r_u) = \frac{k_\mathrm{B}T}{6\pi\eta(r_u)R^{(f)}} \tag{31}$$

and

$$D_t^{(u)}(r_u) = \frac{k_\mathrm{B}T}{6\pi\eta(r_u)R^{(u)}} \tag{32}$$

where the viscosity is a function of r_u. Therefore, rather than assuming eq. (28), one should take into account the effect of the intermediate viscosity:

$$D_t(r_u) = (1 - r_u)D_t^{(f)}(r_u) + r_u D_t^{(u)}(r_u) = (1 - r_u)\frac{\eta^{(f)}}{\eta(r_u)}D_t^{(f)} + r_u\frac{\eta^{(u)}}{\eta(r_u)}D_t^{(u)} \tag{33}$$

If we consider a linear dependence of η on r_u,

$$\eta(r_u) = \eta^{(f)} + r_u(\eta^{(u)} - \eta^{(f)}) \tag{34}$$

with values of $\eta^{(f)}$ and $\eta^{(u)}$ estimated using simulations, we obtain a similar dependence of a_u on r_u as the one based on the actual diffusion coefficients determined from simulations (see Figure S25).

Supplementary Figures

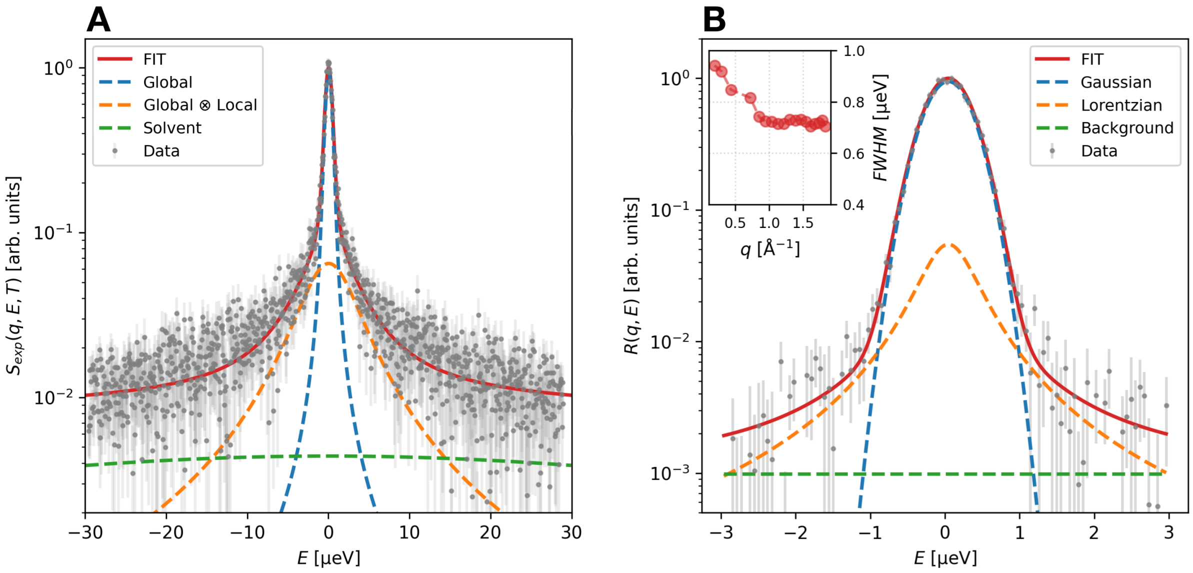

Figure S1: QENS spectra measured at $275K$ and $q = 0.94 \mathring{A}^{-1}$ for the *E. coli* cell pellets (panel A) and the Vanadium (panel B) samples. The red lines in the two graphs represent the result of the best fit of the data with models described in the text, respectively, $S_{exp}(q, E, T)$ and $R(q, E)$. The dashed lines are the different components of the two functions, in particular, for the *E. coli* sample we considered three main diffusive contributions accounting for the global and internal motions of the average protein, and for the D_2O dynamics. The inset in the B panel shows the Full Width Half Maximum (FWHM) of $R(q, E)$, which represents a measure of the energy resolution of the instrument.

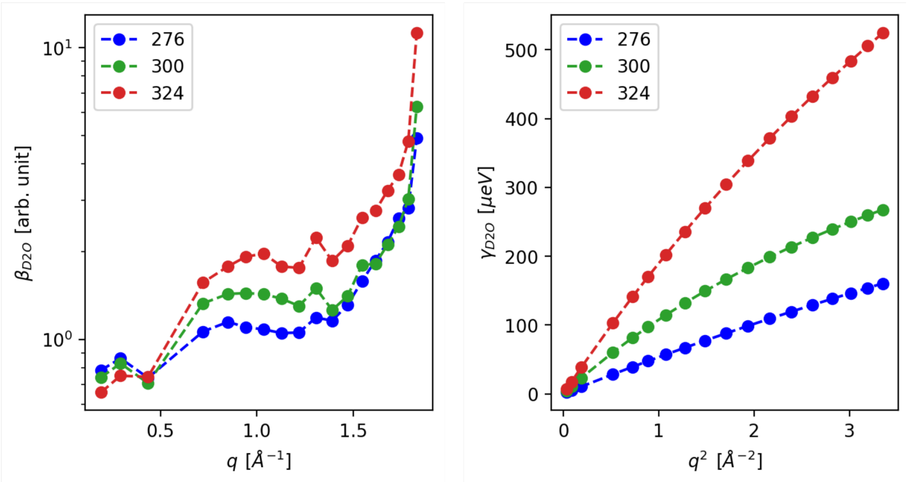

Figure S2: PBS-D_2O buffer at three selected temperatures. Resulting parameters of the fits with the eq. (4).

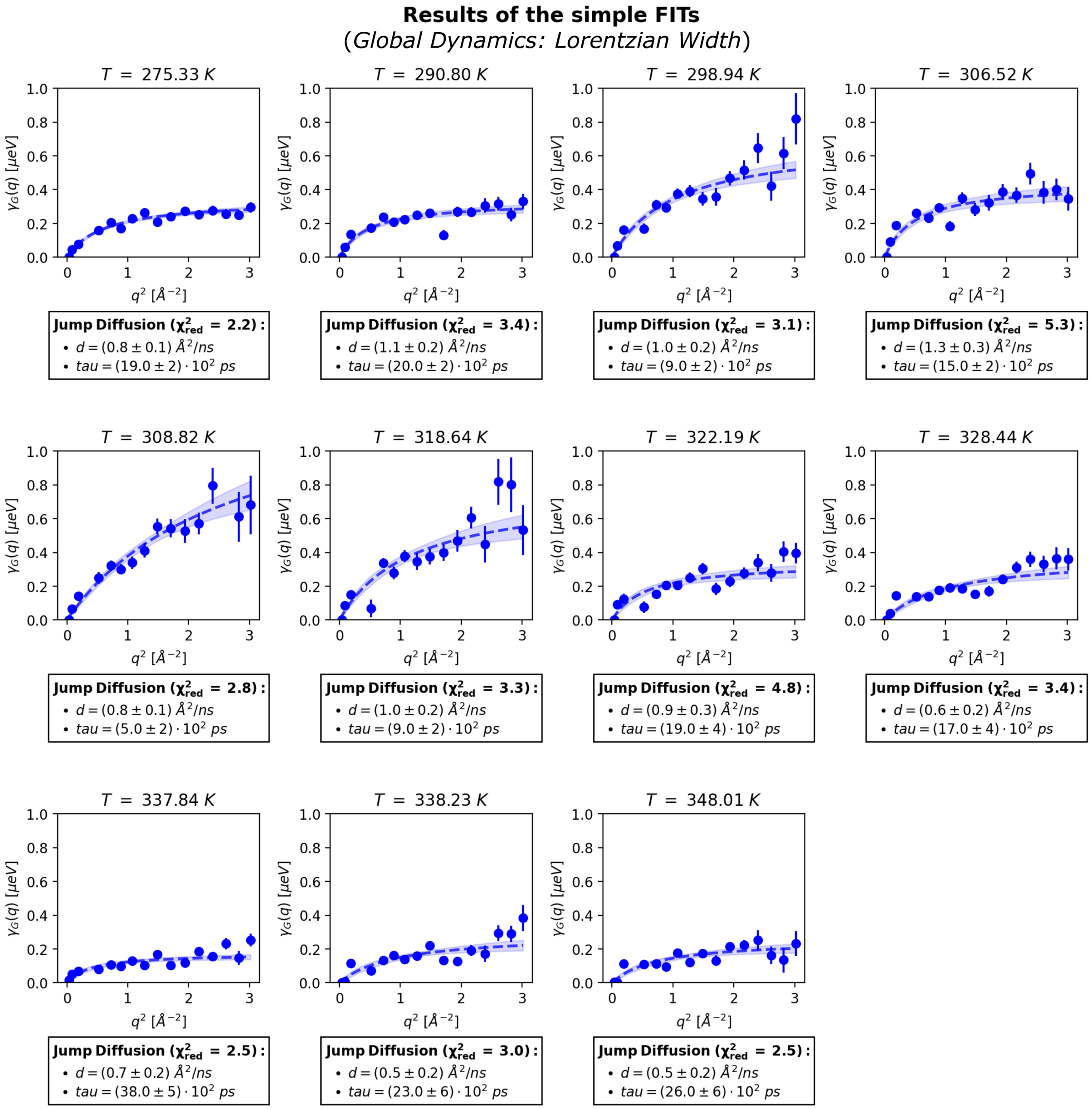

Figure S3: Lorentzian widths γ_G due to global motions of the average protein as function of q^2 for different temperatures. The dashed lines are the results of the fits with the jump-diffusion model described in eq. (6).

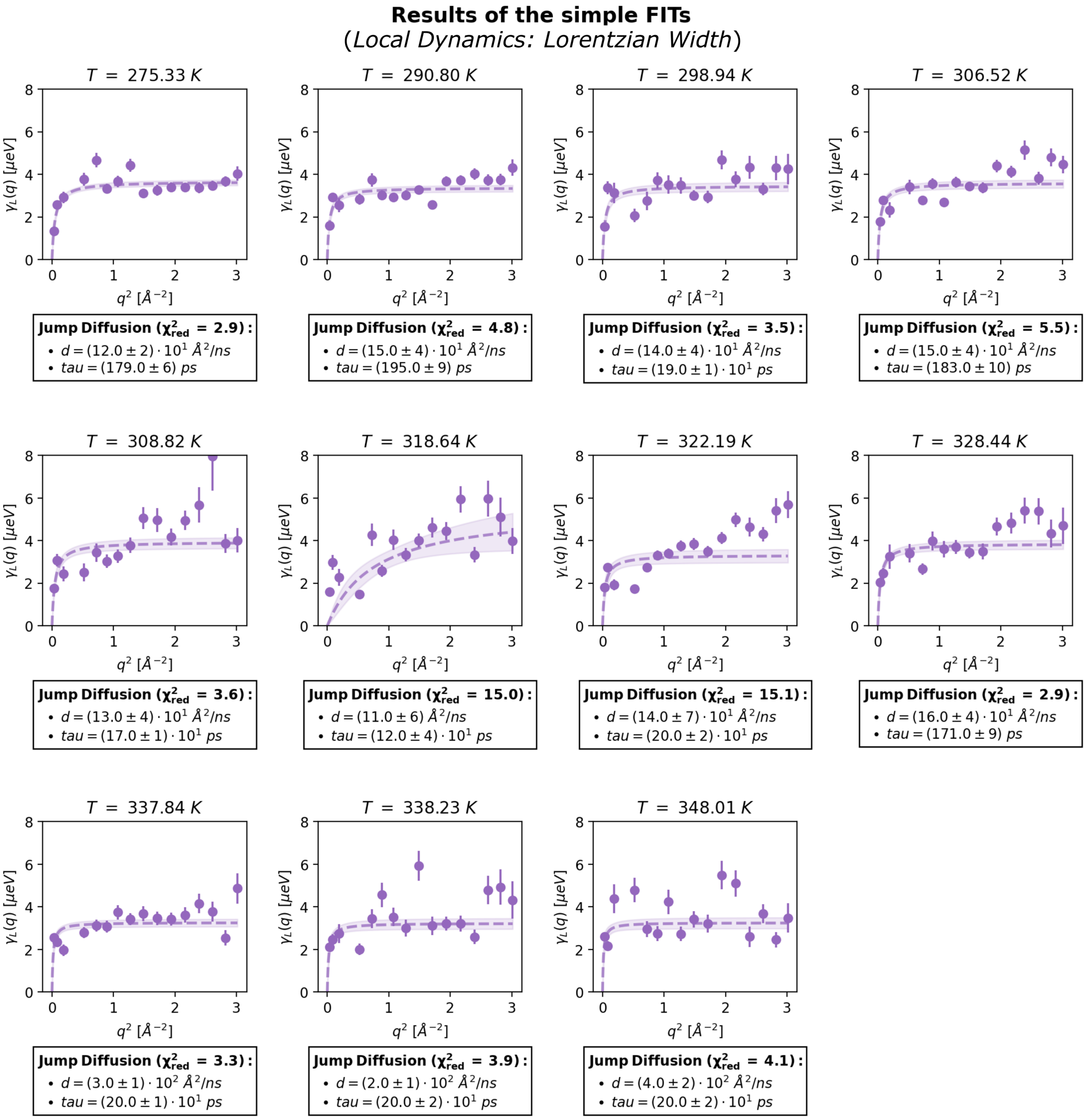

Figure S4: Lorentzian widths γ_L due to local motions of the average protein as function of q^2 for different temperatures. The dashed lines are the results of the fits with the jump-diffusion model described in eq. (6).

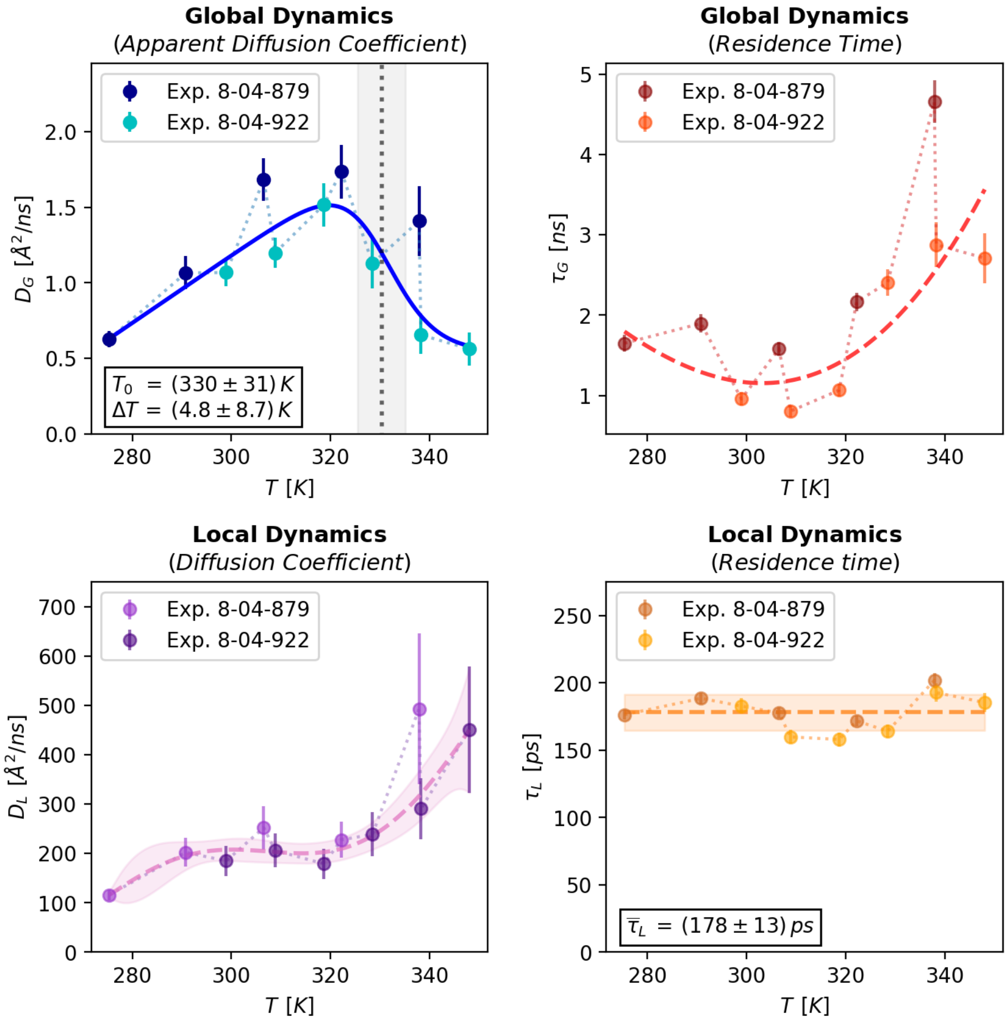

Figure S5: Diffusion coefficients and residence times for the global and local motions obtained from the simultaneous fit of the *E. coli* data, assuming *a priori* the jump-diffusion model eq. (6). The solid blue line is a fit of D_G with the gelation model described by the eq. (7).

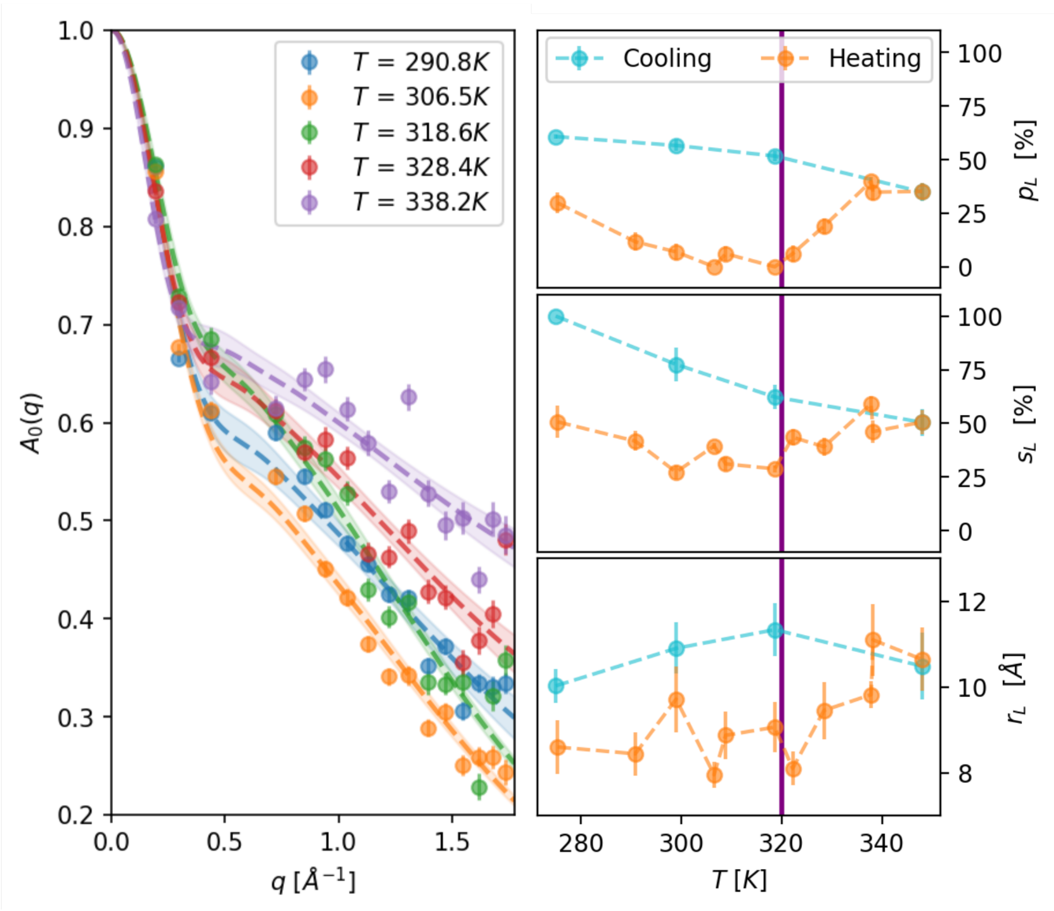

Figure S6: *Left*: EISF derived from the global fit of the *E. coli* data. Dashed lines are the best fits of the EISF with the model described by eq. (9). *Right*: Parameters resulting from the fits of the EISF: p_L is the fraction of H-atoms appearing fixed on the accessible time scale of the instrument, s_L is the fraction of H-atoms diffusing in a spherical volume with radius r_L.

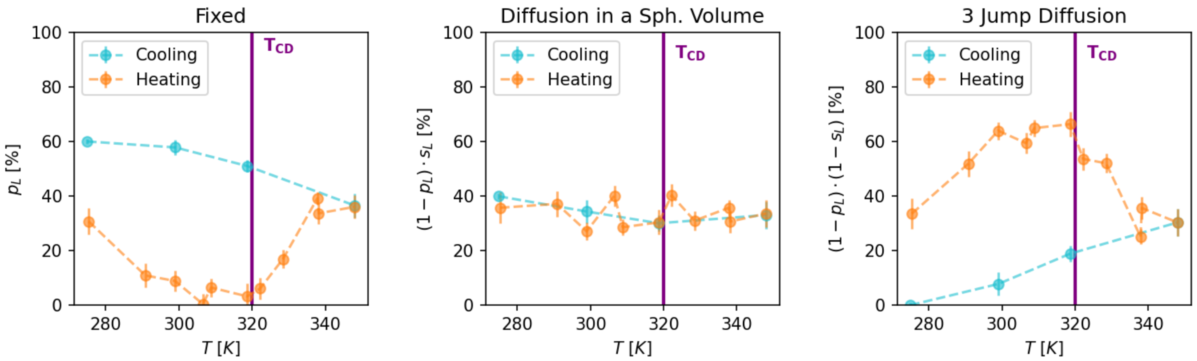

Figure S7: Comparison of the percentage of H-atoms that appear fixed with those H-atoms that diffuse in a spherical volume or undergo a 3-jump diffusion.

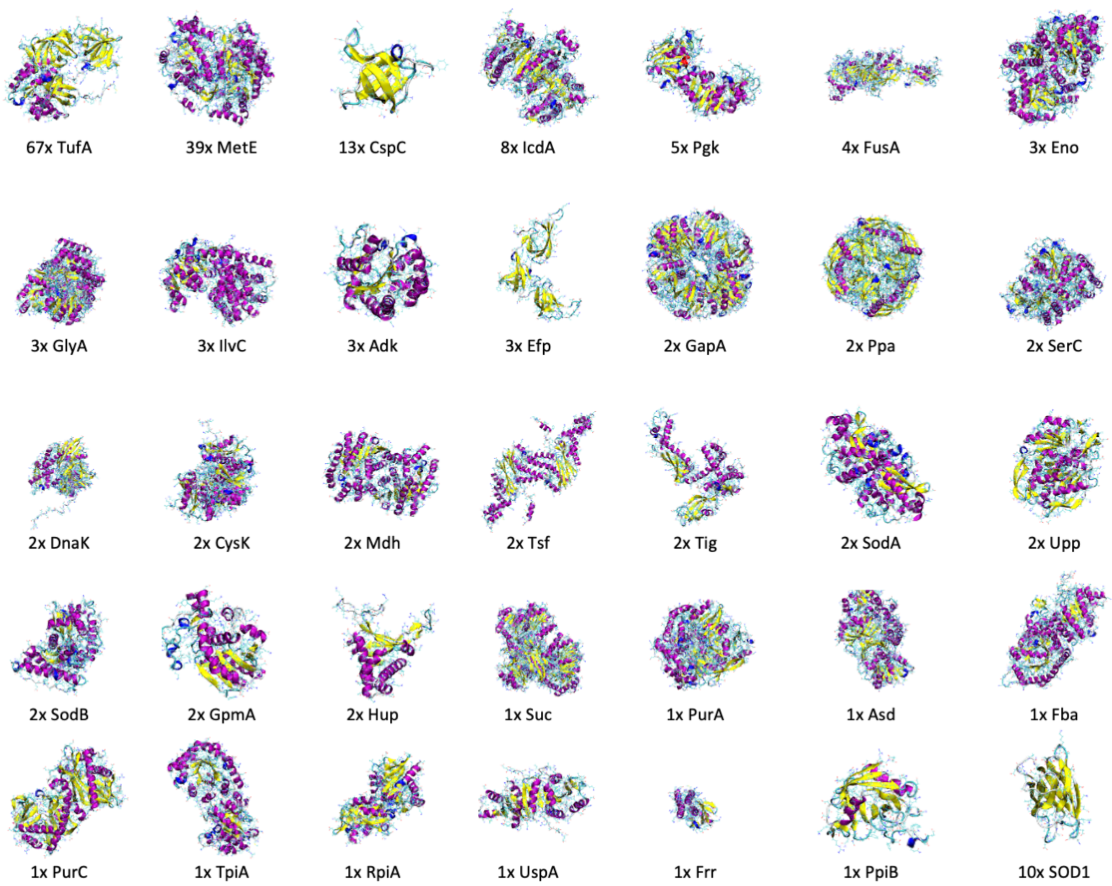

Figure S8: Structures and counts of proteins included in the LBMD simulation. The structure references are given in Table S3.

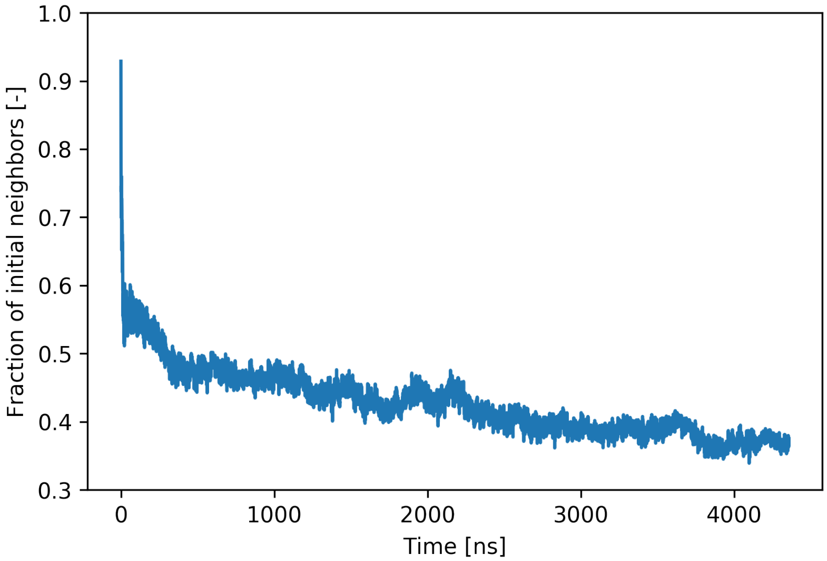

Figure S9: Proportion of initial protein neighbors of an average protein that remain in contact with the protein in the course of the LBMD trajectory. Two proteins were considered in contact if the shortest distance between their beads was below 7.34 Å.

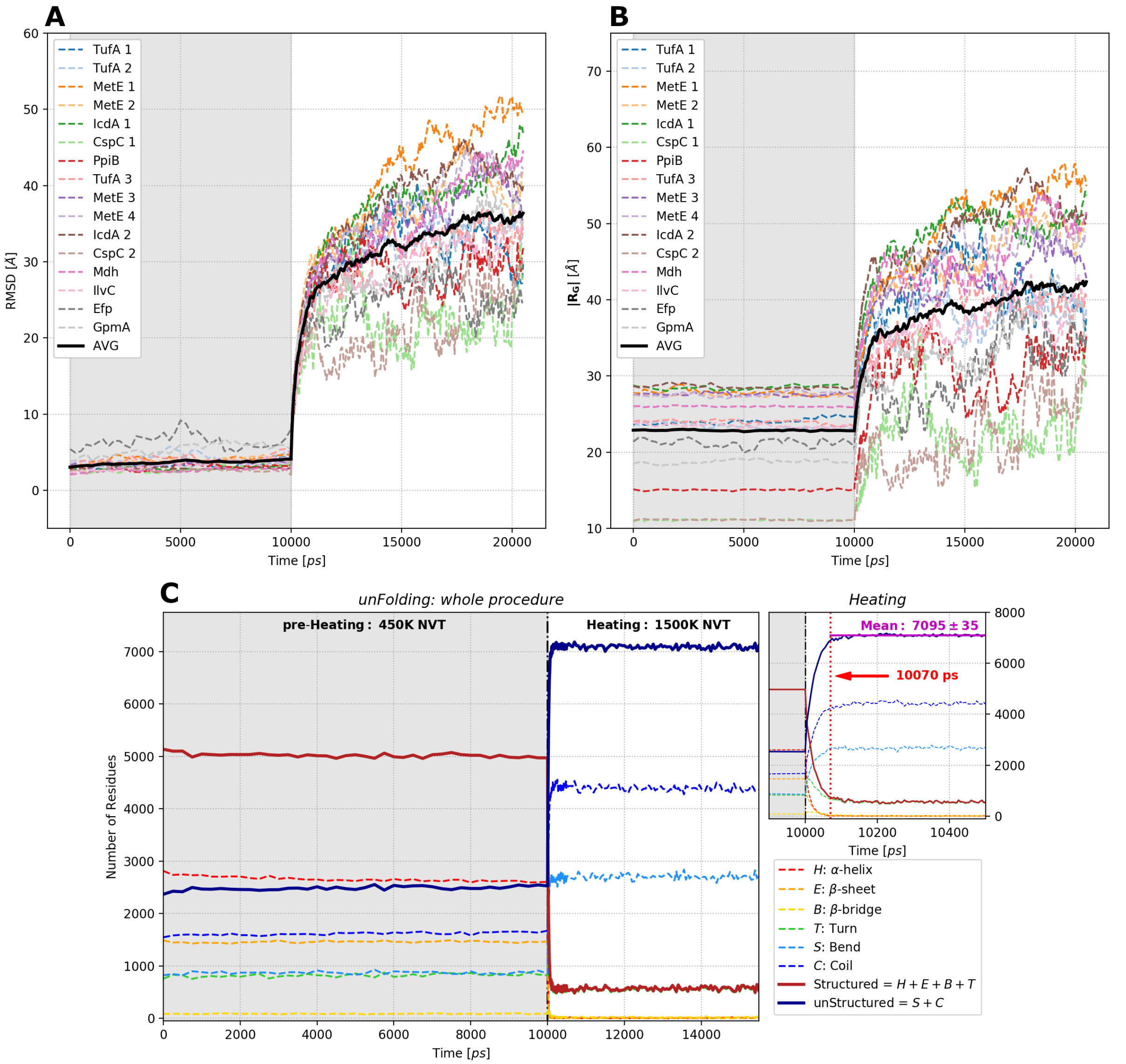

Figure S10: Monitoring the unfolding protocol for each protein in the sub-volume with the concentration of 288 g/L. (A) Root-mean-square deviation (RMSD) of the proteins. (B) Modulus of radius of gyration. (C) Number of residues per secondary structure, as determined by the DSSP software. In the upper right corner, the graph shows a magnification of the main plot on the left to focus on the effects of the heat-shock at 1500 K.

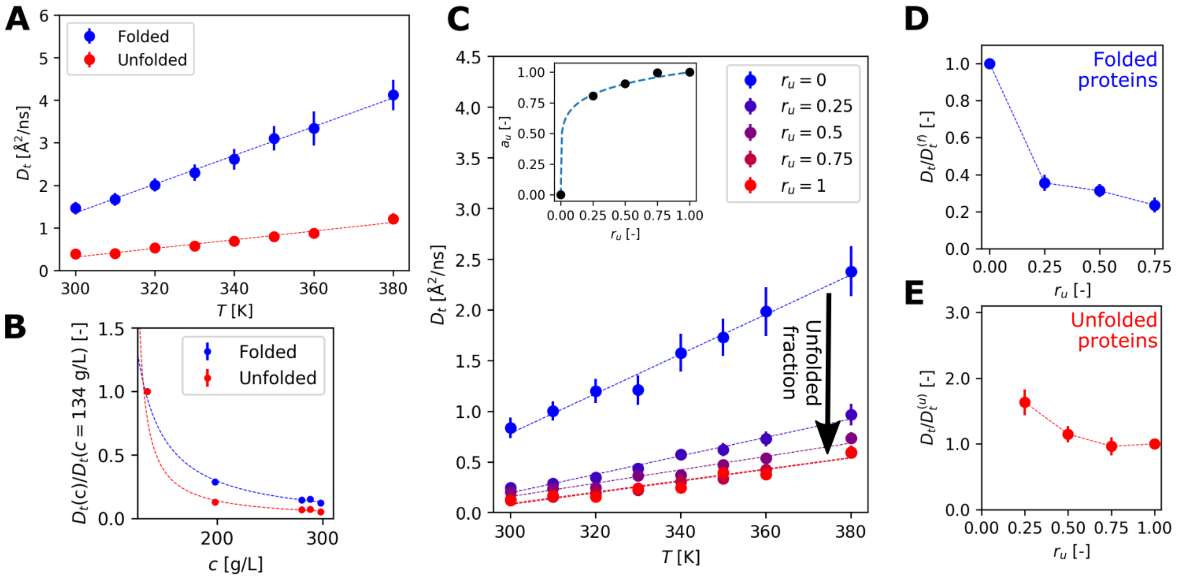

Figure S11: Translational diffusion in sub-volumes described using the CHARMM36m force field. (A) Protein translational diffusion coefficients, computed for the 0.3–5 ns regime. (B) Concentration-dependent diffusion slowdown. The slowdown is expressed relative to the diffusion coefficient for the least concentrated sub-box (134 g/L). Averages of the slowdown values for all the temperatures are shown together with their standard deviations. The dashed lines represent fits with a second-order polynomial fraction. (C) Translational diffusion coefficients in sub-volumes (288 g/L) with a varying fraction of unfolded proteins r_u; The inset shows the dependence—fitted with a power law—of the apparent unfolded fraction a_u on r_u. (D–E) Decrease in the translational diffusion coefficient of folded- and unfolded proteins inside the partially unfolded sub-boxes with increasing r_u.

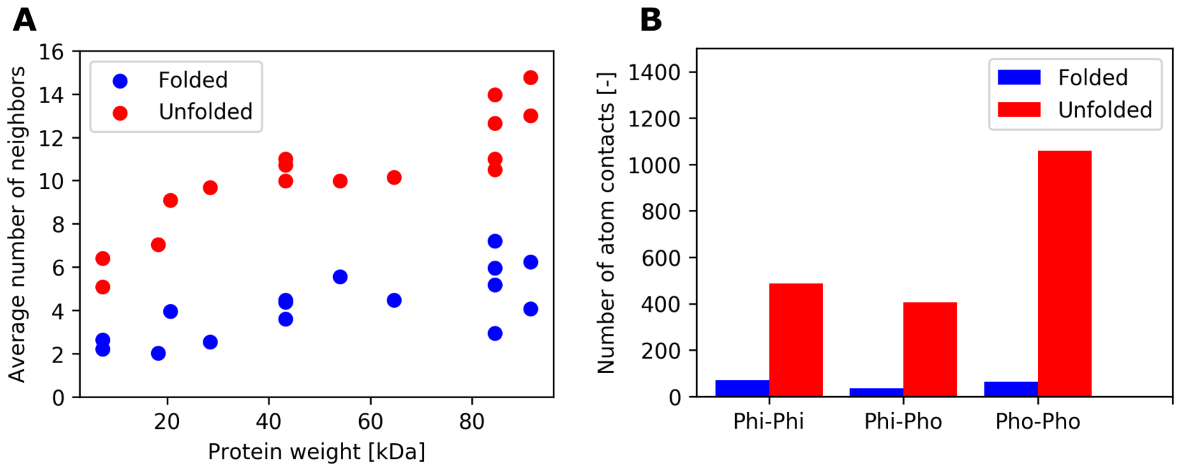

Figure S12: (A) Average number of neighboring proteins per protein molecule as a function of its molecular weight. (B) Average number of different classes of protein–protein contacts (hydrophilic–hydrophilic (Phi–Phi), hydrophilic–hydrophobic (Phi–Pho), and hydrophobic–hydrophobic (Pho–Pho)) per protein molecule. An atom was considered ”hydrophobic” if its partial charge was less than 0.2 e in magnitude. The plots show results obtained from two CHARMM36m trajectories, each extended to 1 μs, of the 288 g/L sub-volume simulated at $T = 330$ K before- and after unfolding.

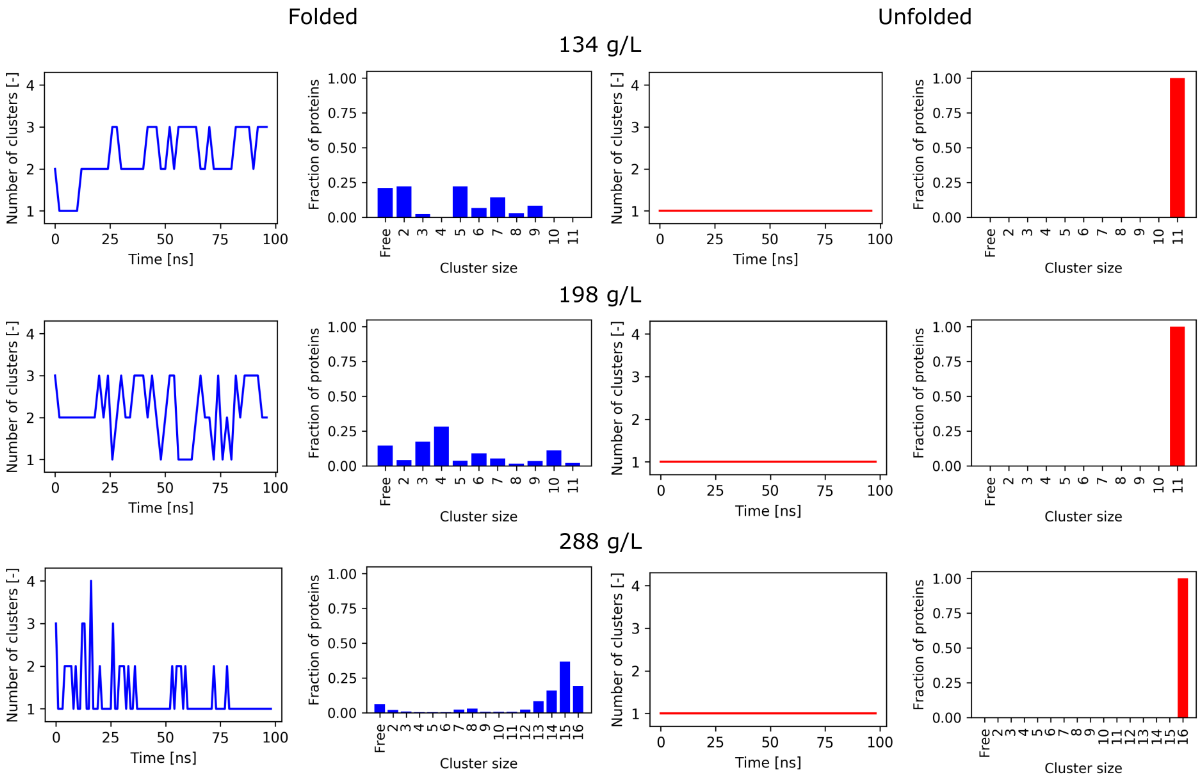

Figure S13: Number of protein clusters present in the course of a sub-volume simulation and the resulting partitioning of proteins into clusters of different sizes for three different protein concentrations in folded (left) and unfolded (right) sub-volumes. The trajectories were obtained with the a99SB-disp force field at $T = 300$ K. Two proteins were considered in contact if the minimum distance between their atoms was lower than 3 Å.

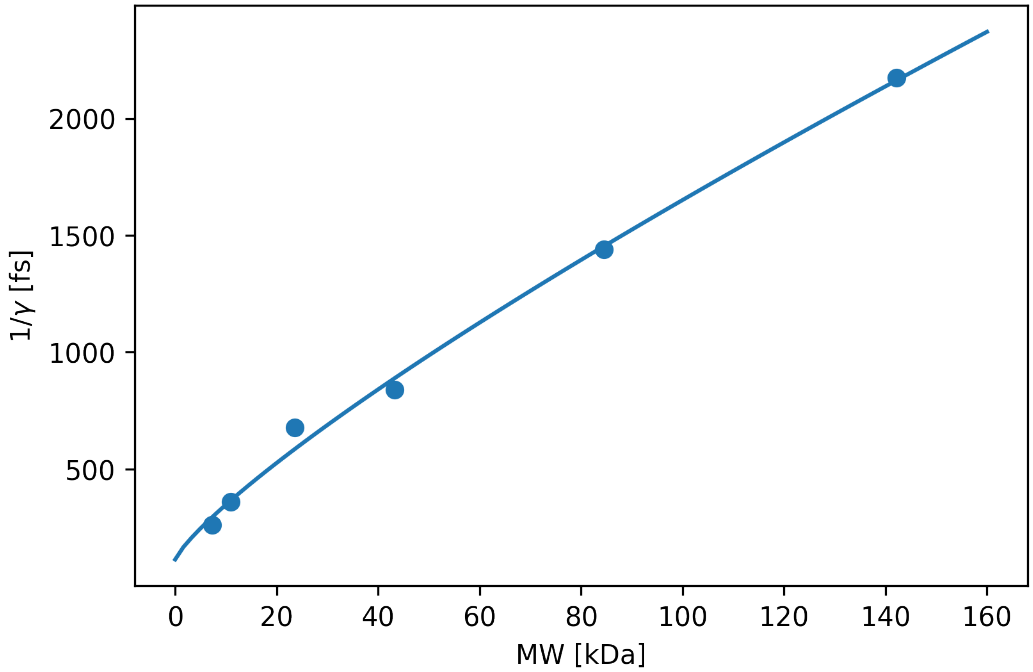

Figure S14: Dependence of the LBMD coupling coefficients γ on protein molecular weight. The γ values for six proteins (CspC, SOD1, Adk, TufA, MetE, and GapA) were optimized in order for the diffusion coefficient of the given isolated protein in an LBMD simulation to match the HYDROPRO (40) prediction. To determine the γ values for the remaining 29 protein species, the resulting dependence of $1/\gamma$ on molecular weight (MW) was fitted with a power-law dependence $1/\gamma = a{\cdot}\mathrm{MW}^b{+}c$, yielding the parameter values $a = 35.76222504$, $b = 0.81676473$, and $c = 113.05584831$ (for MW expressed in kDa and γ in fs^{-1}).

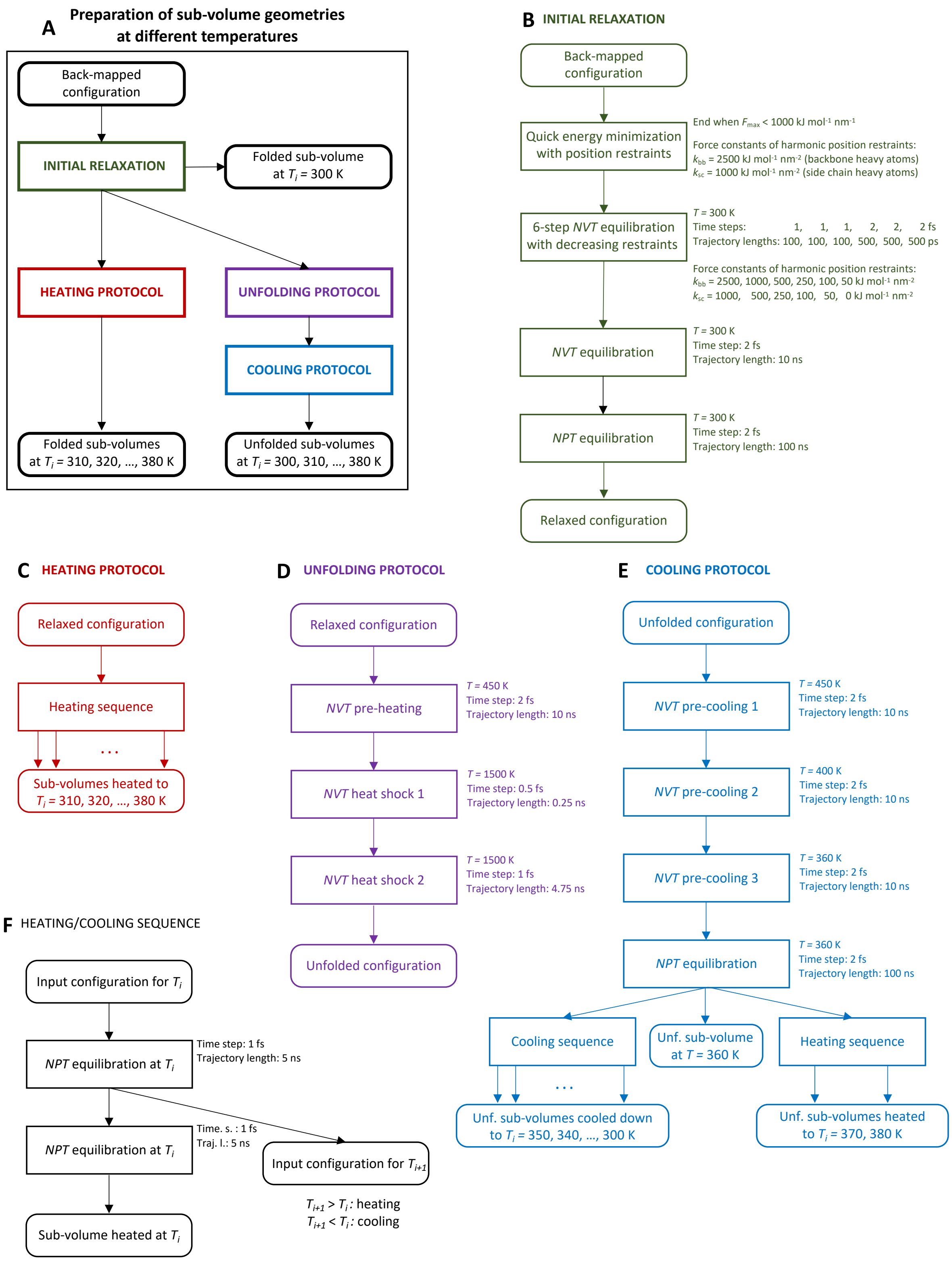

Figure S15: Schematic representation of the procedure used to equilibrate atomistic sub-volumes at different temperatures and in different folding states. (A) General overview of the procedure, (B–F) detailed description of its individual parts.

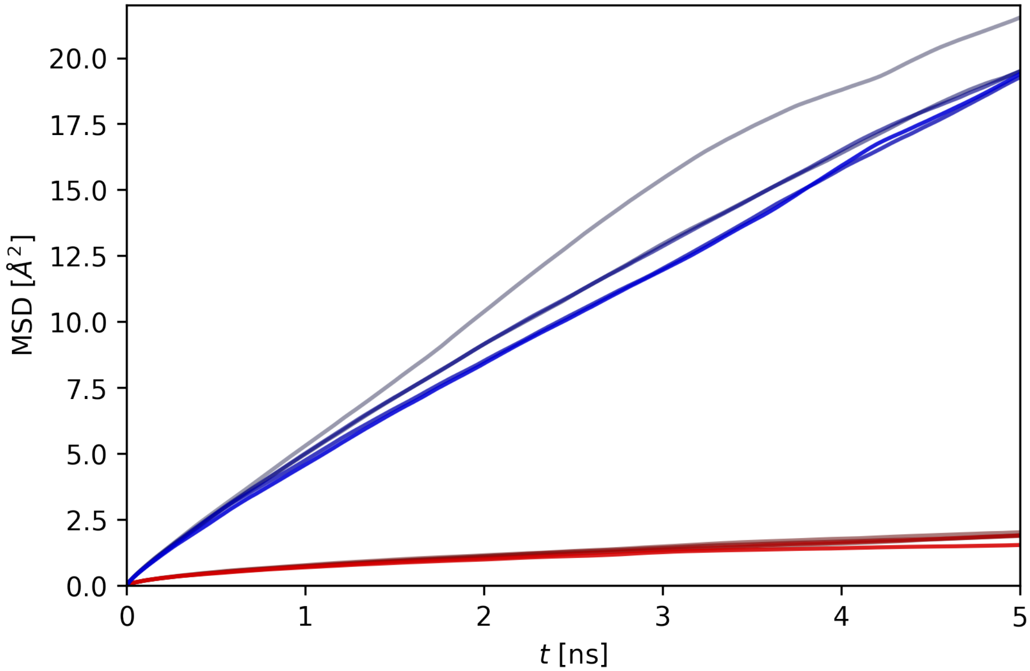

Figure S16: Examples of MSD curves in a folded- (blue) and an unfolded (red) system. The MSD curves were computed as a weighted average over all protein center-of-mass MSDs (see Methods) in 20 ns blocks of a production trajectory performed at $T = 300$ K for the 288 g/L sub-volume using the a99SB-disp force field.

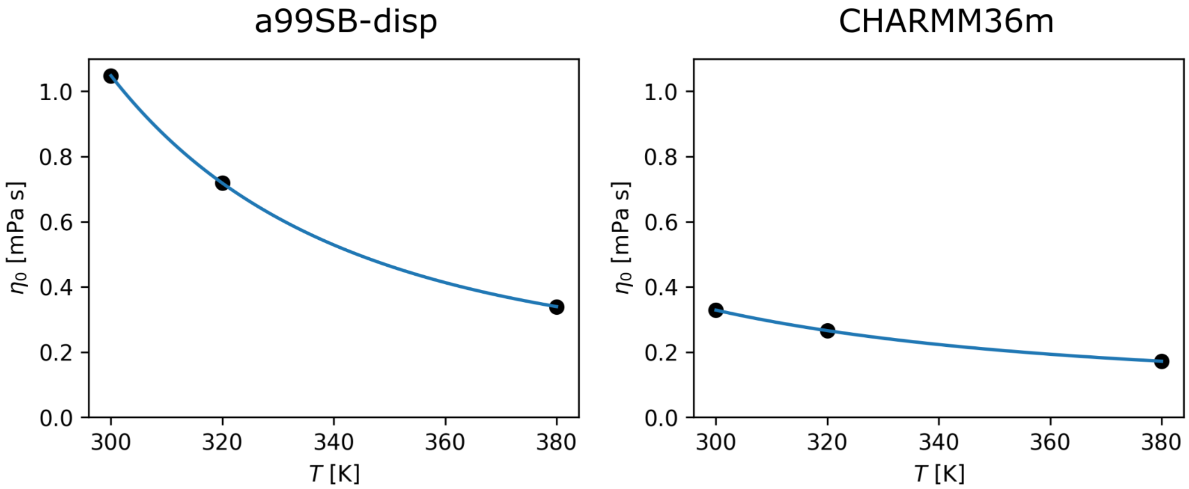

Figure S17: Solvent viscosities determined from simulations. The solvent was modeled as a 150 mM aqueous KCl solution, and the respective water model (a99SB-disp water, or TIP3P) was used. Intermediate viscosity values (blue line) were interpolated using the expression (13).

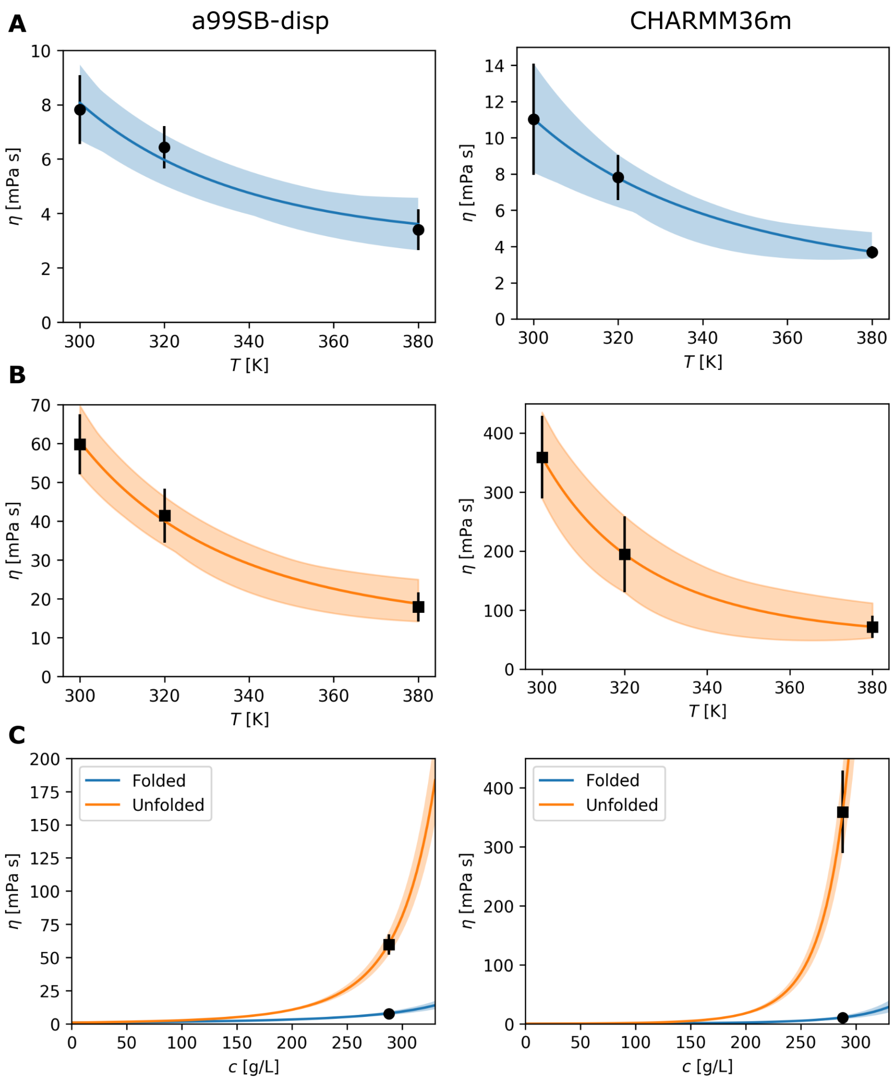

Figure S18: Viscosities calculated for the 288 g/L sub-volume using the a99SB-disp force field (left column) and the CHARMM36m force field (right column). (A) Folded system. (B) Unfolded system. The solid lines show an interpolation using eq. (14) with bands expressing the uncertainty of this interpolation. (C) Extrapolation to different concentrations using eq. (14).

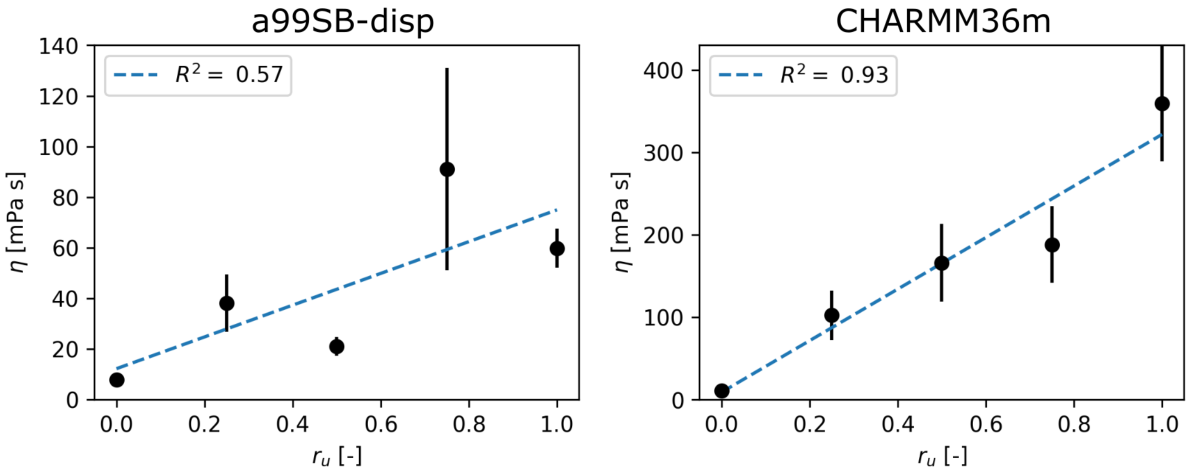

Figure S19: Viscosities calculated for the 288 g/L sub-volume at $T = 300$ K with a varying fraction of unfolded proteins (see Table S6).

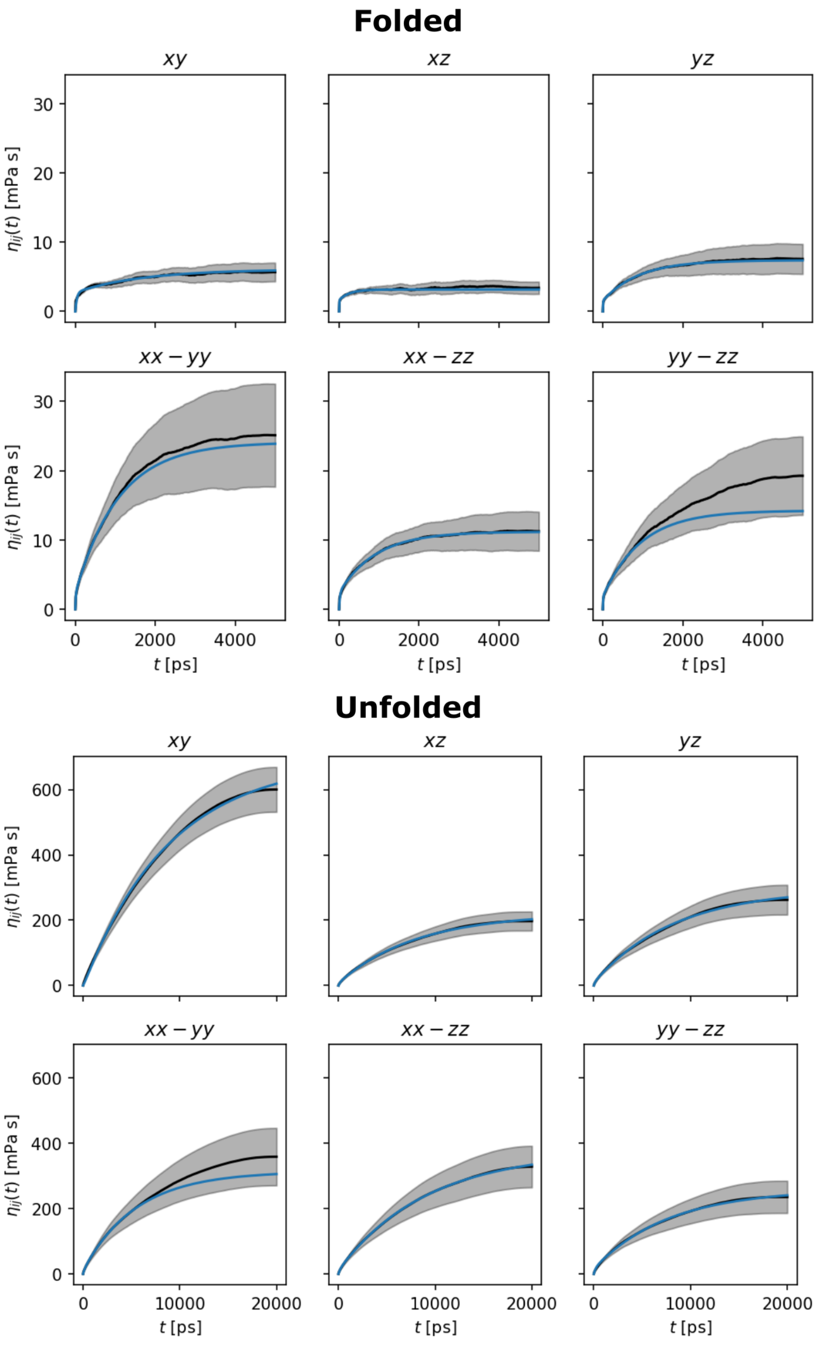

Figure S20: The $\eta_{ij}(t)$ running integrals (eq. (11)) obtained for the 288 g/L CHARMM36m sub-volume at $T = 300$ K. The black curve shows the average of $\eta_{ij}(t)$ over all the NVT simulations, the gray stripe represents the standard error of the mean, and the blue curve is a fit using the analytical function (eq. (12)).

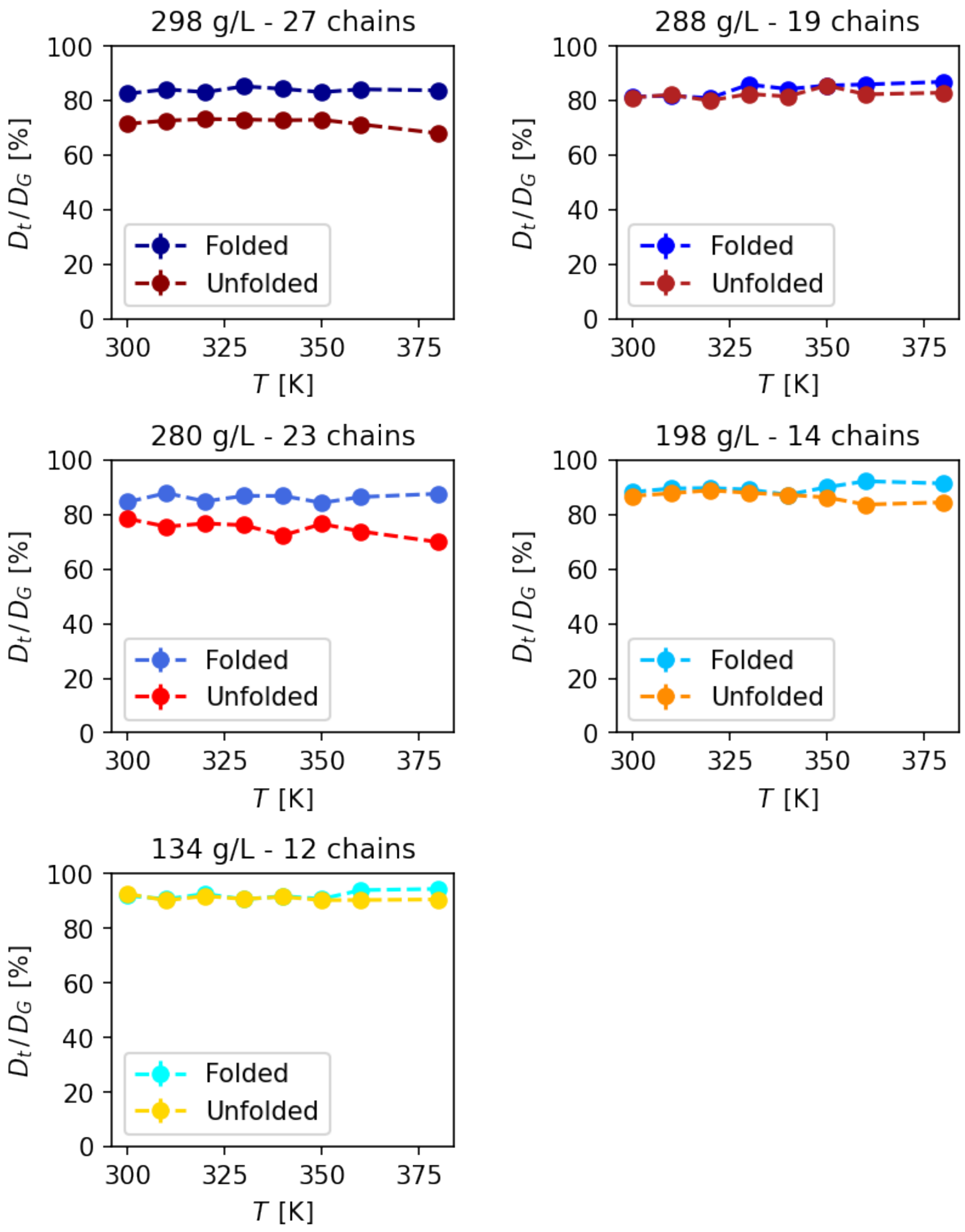

Figure S21: Ratio of the translational and apparent diffusion coefficient for the average protein chain at different temperatures in folded and unfolded sub-volumes with varying protein concentrations (force field: CHARMM36m).

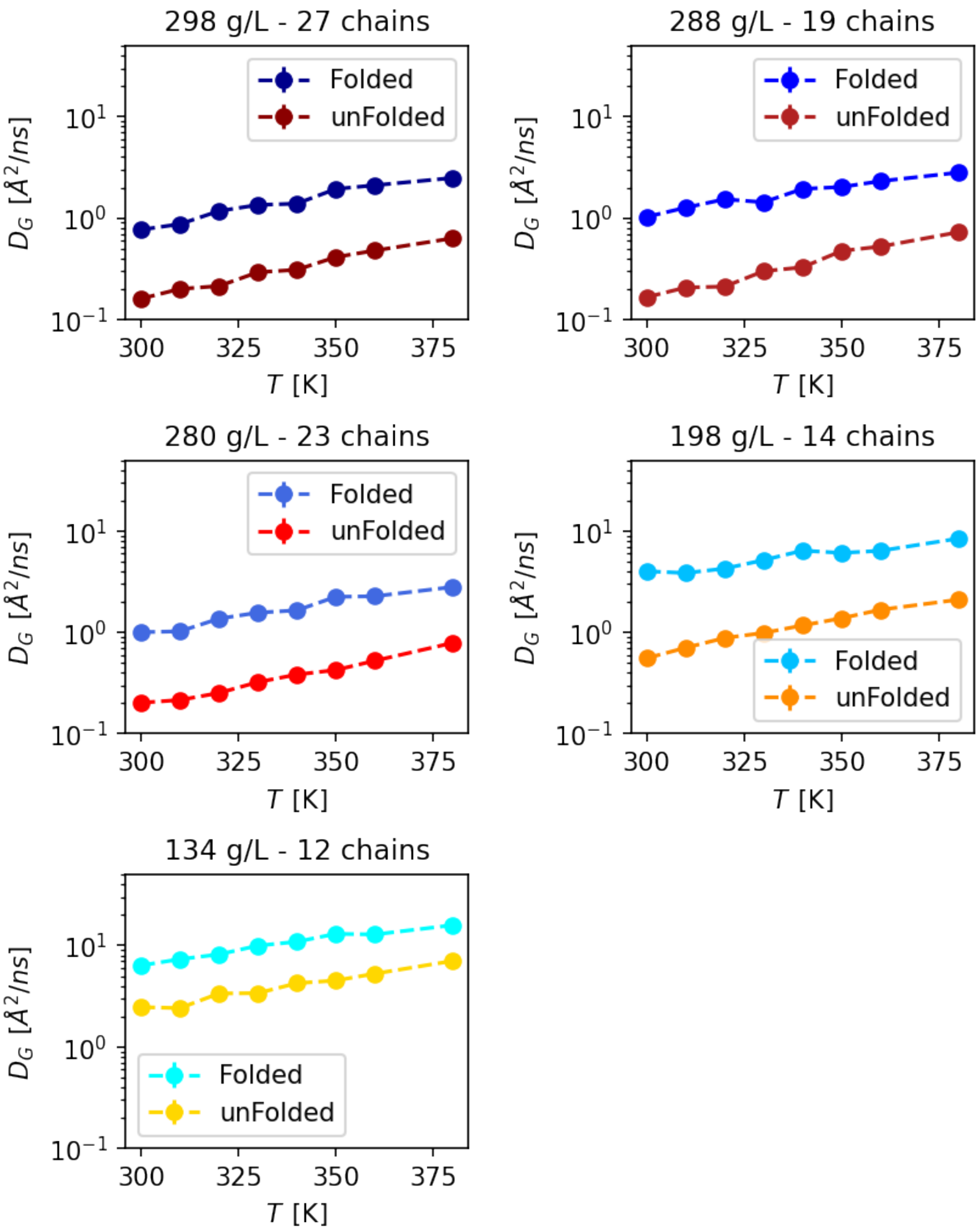

Figure S22: Comparison of the apparent diffusion coefficients for the average folded- and unfolded protein at different temperatures for sub-volumes with different concentrations (force field: CHARMM36m).

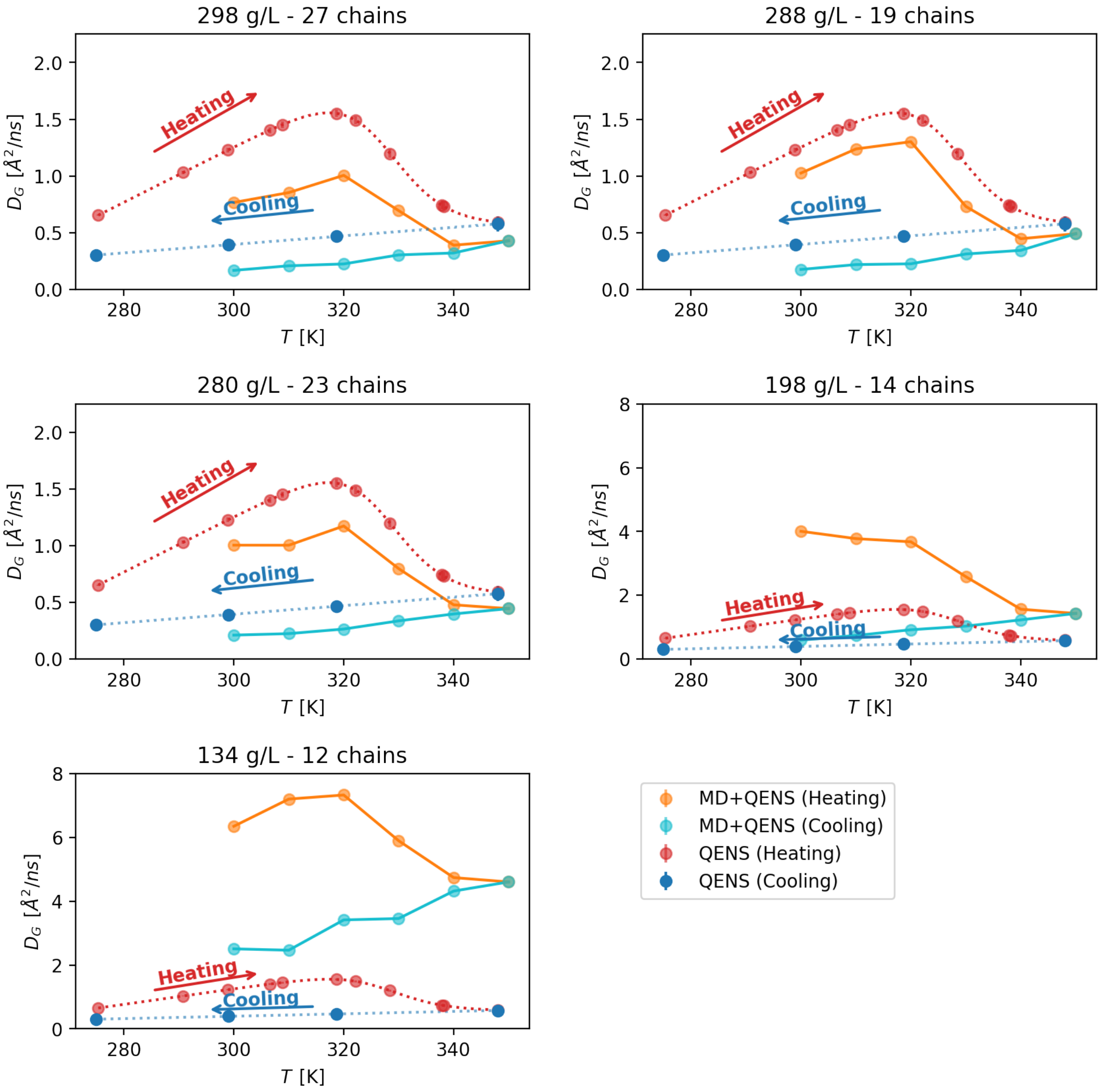

Figure S23: Apparent diffusion coefficient of the average protein at different temperatures for systems with different concentrations (force field: CHARMM36m). The slowdown is reproduced by using the smeared step function a_u^{QENS} to weight the prediction obtained from the simulation of the fully folded and fully unfolded systems.

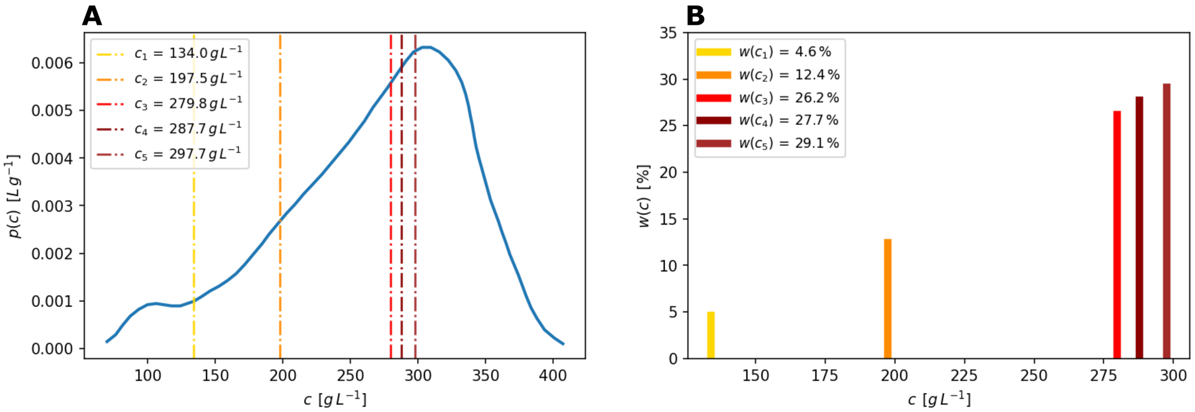

Figure S24: (A) Probability distribution for the selection of a sub-volume of (17 nm)^3 with a concentration c from the larger system with a volume of (40 nm)^3 used in the LBMD simulation for the representation of the *E. coli* cytoplasm. (B) Weight of each sub-volume for the estimation of the apparent diffusion coefficient.

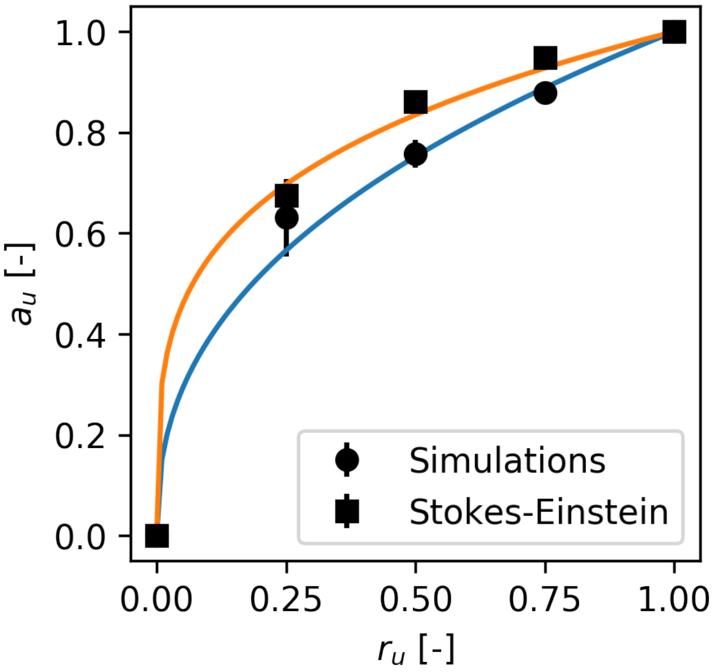

Figure S25: Comparison of the transfer function obtained directly from simulations using the ff99SB-disp force field and a transfer function based on the considerations described in Supplementary Results. Each set of points is fitted with a power law dependence, with the exponents equaling 0.41 and 0.26, respectively.

Supplementary Tables

Table S1: Average values of the parameters resulting from the fit of the Vanadium data with eq. (3).

$A(q)$	(1.003 ± 0.004) arb. units
$F(q)$	0.077 ± 0.013
$E_0(q)$	(0.030 ± 0.003) μeV
$B(q)$	(0.00102 ± 0.00013) arb. units
$\sigma(q)$	(0.317 ± 0.005) μeV
$\gamma(q)$	(0.41 ± 0.08) μeV
FWHM	(0.76 ± 0.07) μeV

Table S2: Parameters shared among all the spectra (i.e. constant in T and q) obtained from the simultaneous fit of the *E. coli* data at all the temperatures with eq. (1), taking into account eq. (2), eq. (4), eq. (5), and assuming *a priori* the models described by eq. (6) and eq. (7).

T_0	(58.7 ± 0.3) C
ΔT	(4.21 ± 0.06) C
a_1	(0.020 ± 0.001) $\mathring{A}^2/(ns \cdot C)$
b_1	(0.605 ± 0.005) $\mathring{A}^2/ns$
a_2	$(10^{-10} \pm 0.001)$ $\mathring{A}^2/(ns \cdot C)$
b_2	(0.48 ± 0.01) $\mathring{A}^2/ns$
ϕ	(40.1 ± 0.1) %

Table S3: Protein composition of the LBMD simulation together with the LBMD coupling coefficient γ for each protein.

	Protein	Structure	MW [kDa]	Count	γ [10^{-3} fs^{-1}]
1	TufA	1DG1	43.3	67	1.1920
2	MetE	SWISS 4zty.1.A	84.5	39	0.6950
3	CspC	SWISS 3i2z.1.B	7.3	13	3.8150
4	IcdA	1P8F	91.5	8	0.6478
5	Pgk	1ZMR	41.1	5	1.1665
6	FusA	4V9O	77.4	4	0.7346
7	Eno	1E9I	91.0	3	0.6504
8	GlyA	1DFO	90.6	3	0.6526
9	IlvC	1YLR	54.1	3	0.9580
10	Adk	1AKE	23.6	3	1.4770
11	Efp	6ENU	20.6	3	1.8653
12	GapA	1S7C	142.1	2	0.4600
13	Ppa	2EIP	117.4	2	0.5357
14	SerC	1BJN	79.3	2	0.7217
15	DnaK	5NRO	69.0	2	0.8008
16	CysK	5J43	68.7	2	0.8031
17	Mdh	2PWZ	64.7	2	0.8400
18	Tsf	1EFU	60.6	2	0.8815
19	Tig	1W26	48.2	2	1.0413
20	SodA	1D5N	45.9	2	1.0779
21	Upp	2EHJ	45.1	2	1.0928
22	SodB	1ISC	42.3	2	1.1438
23	GpmA	1E59	28.4	2	1.5070
24	Hup	2O97	18.8	2	1.9797
25	Suc	1JLL	142.0	1	0.4628
26	PurA	1ADE	94.4	1	0.6326
27	Asd	1BRM	80.0	1	0.7167
28	Fba	5VJE	78.0	1	0.7305
29	PurC	2GQR	54.0	1	0.9591
30	TpiA	4IOT	53.9	1	0.9597
31	RpiA	1KS2	45.5	1	1.0860
32	UspA	SWISS 1jmv.1.A	31.9	1	1.3938
33	Frr	1EK8	20.6	1	1.8626
34	PpiB	2RS4	18.2	1	2.0213
35	SOD1	4BCZ	11.0	10	2.7740

Table S4: Protein concentration and species composition of the five cubic sub-volumes extracted from the LBMD trajectory.

Conc. [g/L]	Protein List
134	CspC (x4), TufA (x3), MetE, Mdh, FusA, SOD1
198	TufA (x4), MetE (x2), CspC, GlyA, CysK, Mdh, SOD1
280	TufA (x2), GapA, GpmA, MetE (x3), CspC, CysK, SodA, IlvC, Hup, DnaK, Frr, SOD1 (x3)
288	TufA (x3), MetE (x4), IcdA (x2), CspC (x2), PpiB, Mdh, IlvC, Efp, GpmA
298	TufA (x8), MetE, CspC (x2), IlvC(x2), SOD1 (x2), Ppa, GlyA, Adk, GpmA, RpiA

Table S5: Overview of an equilibration protocol used for an initial relaxation of isolated protein geometries. k_{BB} and k_{SC} – force constants of harmonic restraint potentials applied to the heavy atoms of the backbone and the side chains, respectively. Δt – time step.

Step	Length [ns]	Δt [fs]	Restraints	k_{BB} [kJ mol^{-1} nm^{-2}]	k_{SC} [kJ mol^{-1} nm^{-2}]
1	0.1	1	heavy atoms	1000	500
2	0.1	1	heavy atoms	500	250
3	0.1	1	heavy atoms	250	100
4	0.5	2	heavy atoms	100	50
5	0.5	2	heavy atoms	50	0
6	0.5	2	none	0	0

Table S6: Protein composition of partially unfolded sub-volumes with a varying fraction r_u of unfolded proteins.

$\mathbf{r_u = 25\ \%}$
UNFOLDED (5):<br>CspC, IlvC, MetE, PpiB, TufA<br><br>FOLDED (11):<br>CspC, Efp, GpmA,2x IcdA, Mdh, 3x MetE 2x TufA

$\mathbf{r_u = 50\ \%}$
UNFOLDED (8):<br>CspC, IcdA, IlvC, 2x MetE, PpiB, 2xTufA<br><br>FOLDED (8):<br>CspC, Efp, GpmA, IcdA, Mdh, 2x MetE, TufA

$\mathbf{r_u = 75\ \%}$
UNFOLDED (11):<br>CspC, 2x IcdA, IlvC, 3x MetE, PpiB, 3x TufA<br><br>FOLDED (5):<br>CspC, Efp, GpmA, Mdh, MetE

Table S7: Parameters resulting from the interpolation of solvent viscosities.

Force field	B_w [-]	D_w [K^{-1}]	ΔE_w [kJ/mol]
a99SB-disp	20.05	0.0115	24.3
CHARMM36m	14.75	0.0054	12.8

Table S8: Parameters describing an analytical dependence derived from the computed viscositites for the crowded protein solution.

Folded

Force field	B [-]	D [K^{-1}]	ΔE [kJ/mol]
a99SB-disp	-20.05	32.8272	7157.1
CHARMM36m	-14.75	12.9697	44982.1

Unfolded

Force field	B [-]	D [K^{-1}]	ΔE [kJ/mol]
a99SB-disp	-20.05	35.2641	36692.3
CHARMM36m	113942.65	189.9209	251039.8

Table S9: Comparison of the estimations obtained with different approaches for the fraction of unfolded proteins $r_u(T)$ in the *E. coli* cytoplasm at increasing temperatures, and the temperature $T_{50\%}$ needed to unfold 50% of the proteins.

**Models**	$r_u(T_{CD})$	$r_u(T_{CD}+3\text{K})$	$r_u(T_{CD}+5\text{K})$	$T_{50\%}$
Dill et al. PNAS 2011	5.39 %	21.47 %	53.54 %	327.9 K
Apparent Fraction	28.75 %	42.65 %	52.72 %	327.6 K
QENS+MD$_{A99d}$	4.82 %	12.59 %	21.07 %	333.0 K
QENS+MD$_{C36m}$	0.02 %	0.25 %	1.07 %	338.7 K

References

[1] Bée, Marc, *Quasielastic neutron scattering.* (United Kingdom: Adam Hilger), (1988).

[2] Jasnin, M and Moulin, M and Haertlein, M and Zaccai, G and Tehei, M, In vivo measurement of internal and global macromolecular motions in Escherichia coli. *Biophysical journal* **95**, 857–864 (2008).

[3] Pounot, Kevin, Ph.D. thesis (Université Grenoble Alpes) (2020).

[4] Grimaldo, Marco and Roosen-Runge, Felix and Jalarvo, Niina and Zamponi, Michaela and Zanini, Fabio and Hennig, Marcus and Zhang, Fajun and Schreiber, Frank and Seydel, Tilo, High-resolution neutron spectroscopy on protein solution samples in *EPJ Web of Conferences.* (EDP Sciences), Vol. 83, p. 02005 (2015).

[5] Vineyard, George H, Scattering of slow neutrons by a liquid. *Physical Review* **110**, 999 (1958).

[6] Wanderlingh, U and D'Angelo, G and Branca, C and Conti Nibali, V and Trimarchi, A and Rifici, S and Finocchiaro, D and Crupi, C and Ollivier, J and Middendorf, HD, Multi-component modeling of quasielastic neutron scattering from phospholipid membranes. *The Journal of chemical physics* **140**, 05B602_1 (2014).

[7] Bosio, L and Teixeira, J and Bellissent-Funel, M-C, Enhanced density fluctuations in water analyzed by neutron scattering. *Physical Review A* **39**, 6612 (1989).

[8] Grimaldo, Marco and Roosen-Runge, Felix and Hennig, Marcus and Zanini, Fabio and Zhang, Fajun and Jalarvo, Niina and Zamponi, Michaela and Schreiber, Frank and Seydel, Tilo, Hierarchical molecular dynamics of bovine serum albumin in concentrated aqueous solution below and above thermal denaturation. *Physical Chemistry Chemical Physics* **17**, 4645–4655 (2015).

[9] Martinez, Nicolas and Michoud, Gregoire and Cario, A and Ollivier, J and Franzetti, Bruno and Jebbar, Mohamed and Oger, P and Peters, Judith, High protein flexibility and reduced hydration water dynamics are key pressure adaptive strategies in prokaryotes. *Scientific reports* **6**, 1–11 (2016).

[10] Mamontov, Eugene, Microscopic diffusion processes measured in living planarians. *Scientific reports* **8**, 1–8 (2018).

[11] Singwi, KS and Sjölander, Alf, Diffusive motions in water and cold neutron scattering. *Physical Review* **119**, 863 (1960).

[12] Hennig, Marcus and Roosen-Runge, Felix and Zhang, Fajun and Zorn, Stefan and Skoda, Maximilian WA and Jacobs, Robert MJ and Seydel, Tilo and Schreiber, Frank, Dynamics of highly concentrated protein solutions around the denaturing transition. *Soft Matter* **8**, 1628–1633 (2012).

[13] Grimaldo, Marco and Roosen-Runge, Felix and Zhang, Fajun and Seydel, Tilo and Schreiber, Frank, Diffusion and dynamics of γ-globulin in crowded aqueous solutions. *The Journal of Physical Chemistry B* **118**, 7203–7209 (2014).

[14] McGuffee, Sean R and Elcock, Adrian H, Diffusion, crowding & protein stability in a dynamic molecular model of the bacterial cytoplasm. *PLoS computational biology* **6**, e1000694 (2010).

[15] Link, Andrew J and Robison, Keith and Church, George M, Comparing the predicted and observed properties of proteins encoded in the genome of Escherichia coli K-12. *Electrophoresis* **18**, 1259–1313 (1997).

[16] Danielsson, Jens and Awad, Wael and Saraboji, Kadhirvel and Kurnik, Martin and Lang, Lisa and Leinartaitė, Lina and Marklund, Stefan L and Logan, Derek T and Oliveberg, Mikael, Global structural motions from the strain of a single hydrogen bond. *Proceedings of the National Academy of Sciences* **110**, 3829–3834 (2013).

[17] Berman, Helen and Henrick, Kim and Nakamura, Haruki, Announcing the worldwide protein data bank. *Nature Structural @AND@ Molecular Biology* **10**, 980–980 (2003).

[18] Waterhouse, Andrew and Bertoni, Martino and Bienert, Stefan and Studer, Gabriel and Tauriello, Gerardo and Gumienny, Rafal and Heer, Florian T and de Beer, Tjaart A P and Rempfer, Christine and Bordoli, Lorenza and others, SWISS-MODEL: homology modelling of protein structures and complexes. *Nucleic acids research* **46**, W296–W303 (2018).

[19] , Andrej and Blundell, Tom L, Comparative protein modelling by satisfaction of spatial restraints. *Journal of molecular biology* **234**, 779–815 (1993).

[20] Williams, Christopher J and Headd, Jeffrey J and Moriarty, Nigel W and Prisant, Michael G and Videau, Lizbeth L and Deis, Lindsay N and Verma, Vishal and Keedy, Daniel A and Hintze, Bradley J and Chen, Vincent B and others, MolProbity: More and better reference data for improved all-atom structure validation. *Protein Science* **27**, 293–315 (2018).

[21] Krissinel, Evgeny and Henrick, Kim, Inference of macromolecular assemblies from crystalline state. *Journal of molecular biology* **372**, 774–797 (2007).

[22] Wiborg, Ove and Andersen, Carsten and Knudsen, Charlotte R and Clark, Brian FC and Nyborg, Jens, Mapping Escherichia coli elongation factor Tu residues involved in binding of aminoacyl-tRNA. *Journal of Biological Chemistry* **271**, 20406–20411 (1996).

[23] Keseler, Ingrid M and Collado-Vides, Julio and Santos-Zavaleta, Alberto and Peralta-Gil, Martin and Gama-Castro, Socorro and Muñiz-Rascado, Luis and Bonavides-Martinez, César and Paley, Suzanne and Krummenacker, Markus and Altman, Tomer and others, EcoCyc: a comprehensive database of Escherichia coli biology. *Nucleic acids research* **39**, D583–D590 (2010).

[24] Abraham, Mark James and Murtola, Teemu and Schulz, Roland and Páll, Szilárd and Smith, Jeremy C and Hess, Berk and Lindahl, Erik, GROMACS: High performance molecular simulations through multi-level parallelism from laptops to supercomputers. *SoftwareX* **1**, 19–25 (2015).

[25] Robustelli, Paul and Piana, Stefano and Shaw, David E, Developing a molecular dynamics force field for both folded and disordered protein states. *Proceedings of the National Academy of Sciences* **115**, E4758–E4766 (2018).

[26] Huang, Jing and Rauscher, Sarah and Nawrocki, Grzegorz and Ran, Ting and Feig, Michael and De Groot, Bert L and Grubmüller, Helmut and MacKerell, Alexander D, CHARMM36m: an improved force field for folded and intrinsically disordered proteins. *Nature methods* **14**, 71–73 (2017).

[27] Berendsen, Herman JC and Postma, JPM van and van Gunsteren, Wilfred F and DiNola, ARHJ and Haak, Jan R, Molecular dynamics with coupling to an external bath. *The Journal of chemical physics* **81**, 3684–3690 (1984).

[28] Mart´, Leandro and Andrade, Ricardo and Birgin, Ernesto G and Mart´, José Mario, PACKMOL: a package for building initial configurations for molecular dynamics simulations. *Journal of computational chemistry* **30**, 2157–2164 (2009).

[29] Ahlrichs, Patrick and Dünweg, Burkhard, Simulation of a single polymer chain in solution by combining lattice Boltzmann and molecular dynamics. *Journal of Chemical Physics* **111**, 8225–8239 (1999).

[30] Sterpone, Fabio and Derreumaux, Philippe and Melchionna, Simone, Protein simulations in fluids: Coupling the OPEP coarse-grained force field with hydrodynamics. *Journal of chemical theory and computation* **11**, 1843–1853 (2015).

[31] Bernaschi, Massimo and Melchionna, Simone and Succi, Sauro and Fyta, Maria and Kaxiras, Efthimios and Sircar, Joy K, MUPHY: A parallel MUlti PHYsics/scale code for high performance bio-fluidic simulations. *Computer Physics Communications* **180**, 1495–1502 (2009).

[32] Chiricotto, Mara and Melchionna, Simone and Derreumaux, Philippe and Sterpone, Fabio, Hydrodynamic effects on β-amyloid (16-22) peptide aggregation. *The Journal of chemical physics* **145**, 035102 (2016).

[33] Chiricotto, Mara and Melchionna, Simone and Derreumaux, Philippe and Sterpone, Fabio, Multiscale aggregation of the amyloid Aβ16–22 peptide: from disordered coagulation and lateral branching to Amorphous prefibrils. *The journal of physical chemistry letters* **10**, 1594–1599 (2019).

[34] Sterpone, Fabio and Derreumaux, Philippe and Melchionna, Simone, Molecular mechanism of protein unfolding under shear: A lattice boltzmann molecular dynamics study. *The Journal of Physical Chemistry B* **122**, 1573–1579 (2018).

[35] Brandner, Astrid F and Timr, Stepan and Melchionna, Simone and Derreumaux, Philippe and Baaden, Marc and Sterpone, Fabio, Modelling lipid systems in fluid with Lattice Boltzmann Molecular Dynamics simulations and hydrodynamics. *Scientific reports* **9** (2019).

[36] Sterpone, Fabio and Melchionna, Simone and Tuffery, Pierre and Pasquali, Samuela and Mousseau, Normand and Cragnolini, Tristan and Chebaro, Yassmine and St-Pierre, Jean-Francois and Kalimeri, Maria and Barducci, Alessandro and others, The OPEP protein model: from single molecules, amyloid formation, crowding and hydrodynamics to DNA/RNA systems. *Chemical Society reviews* **43**, 4871–4893 (2014).

[37] Timr, Stepan and Sterpone, Fabio, Stabilizing or Destabilizing: Simulations of Chymotrypsin Inhibitor 2 under Crowding Reveal Existence of a Crossover Temperature. *The Journal of Physical Chemistry Letters* **12**, 1741–1746 (2021).

[38] Succi, Sauro, *The lattice Boltzmann equation: for fluid dynamics and beyond.* (Oxford university press), (2001).

[39] Benzi, Roberto and Succi, Sauro and Vergassola, Massimo, The lattice Boltzmann equation: theory and applications. *Physics Reports* **222**, 145–197 (1992).

[40] Ortega, Alvaro and Amorós, D and De La Torre, J Garc´, Prediction of hydrodynamic and other solution properties of rigid proteins from atomic-and residue-level models. *Biophysical journal* **101**, 892–898 (2011).

[41] Jorgensen, William L and Chandrasekhar, Jayaraman and Madura, Jeffry D and Impey, Roger W and Klein, Michael L, Comparison of simple potential functions for simulating liquid water. *The Journal of chemical physics* **79**, 926–935 (1983).

[42] Darden, Tom and York, Darrin and Pedersen, Lee, Particle mesh Ewald: An N log(N) method for Ewald sums in large systems. *The Journal of chemical physics* **98**, 10089–10092 (1993).

[43] Hockney, Roger Williams and Goel, SP and Eastwood, JW, Quiet high-resolution computer models of a plasma. *Journal of Computational Physics* **14**, 148–158 (1974).

[44] Hess, Berk and Bekker, Henk and Berendsen, Herman JC and Fraaije, Johannes GEM, LINCS: a linear constraint solver for molecular simulations. *Journal of computational chemistry* **18**, 1463–1472 (1997).

[45] Miyamoto, Shuichi and Kollman, Peter A, Settle: An analytical version of the SHAKE and RATTLE algorithm for rigid water models. *Journal of computational chemistry* **13**, 952–962 (1992).

[46] Bussi, Giovanni and Donadio, Davide and Parrinello, Michele, Canonical sampling through velocity rescaling. *The Journal of chemical physics* **126**, 014101 (2007).

[47] Parrinello, Michele and Rahman, Aneesur, Polymorphic transitions in single crystals: A new molecular dynamics method. *Journal of Applied physics* **52**, 7182–7190 (1981).

[48] Seddon, Gavin M and Bywater, Robert P, Accelerated simulation of unfolding and refolding of a large single chain globular protein. *Open Biology* **2**, 120087 (2012).

[49] Touw, Wouter G and Baakman, Coos and Black, Jon and Te Beek, Tim AH and Krieger, Elmar and Joosten, Robbie P and Vriend, Gert, A series of PDB-related databanks for everyday needs. *Nucleic acids research* **43**, D364–D368 (2015).

[50] Kabsch, Wolfgang and Sander, Christian, Dictionary of protein secondary structure: pattern recognition of hydrogen-bonded and geometrical features. *Biopolymers: Original Research on Biomolecules* **22**, 2577–2637 (1983).

[51] Wong, Vance and Case, David A., Evaluating rotational diffusion from protein MD simulations. *Journal of Physical Chemistry B* **112**, 6013–6024 (2008).

[52] Nawrocki, Grzegorz and Wang, Po-hung and Yu, Isseki and Sugita, Yuji and Feig, Michael, Slow-Down in Diffusion in Crowded Protein Solutions Correlates with Transient Cluster Formation. *The Journal of Physical Chemistry B* **121**, 11072–11084 (2017).

[53] von Bülow, Sören and Siggel, Marc and Linke, Max and Hummer, Gerhard, Dynamic cluster formation determines viscosity and diffusion in dense protein solutions. *Proceedings of the National Academy of Sciences* **116**, 9843–9852 (2019).

[54] Monkos, K., Viscosity of bovine serum albumin aqueous solutions as a function of temperature and concentration. *International Journal of Biological Macromolecules* **18**, 61–68 (1996).

[55] Yeh, In Chul and Hummer, Gerhard, System-size dependence of diffusion coefficients and viscosities from molecular dynamics simulations with periodic boundary conditions. *Journal of Physical Chemistry B* **108**, 15873–15879 (2004).

[56] Linke, Max and Köfinger, Jürgen and Hummer, Gerhard, Rotational Diffusion Depends on Box Size in Molecular Dynamics Simulations. *Journal of Physical Chemistry Letters* **9**, 2874–2878 (2018).

[57] Roosen-Runge, Felix and Hennig, Marcus and Zhang, Fajun and Jacobs, Robert MJ and Sztucki, Michael and Schober, Helmut and Seydel, Tilo and Schreiber, Frank, Protein self-diffusion in crowded solutions. *Proceedings of the National Academy of Sciences* **108**, 11815–11820 (2011).
